# The impact of quality-adjusted life years on evaluating COVID-19 mitigation strategies: lessons from age-specific vaccination roll-out and variants of concern in Belgium (2020-2022)

**DOI:** 10.1186/s12889-024-18576-w

**Published:** 2024-04-26

**Authors:** Lander Willem, Steven Abrams, Nicolas Franco, Pietro Coletti, Pieter J. K. Libin, James Wambua, Simon Couvreur, Emmanuel André, Tom Wenseleers, Zhuxin Mao, Andrea Torneri, Christel Faes, Philippe Beutels, Niel Hens

**Affiliations:** 1 Department of Family Medicine and Population Health, Antwerp, Belgium; 2 Centre for Health Economic Research and Modelling Infectious Diseases, University of Antwerp, Antwerp, Belgium; 3 Data Science Institute, Hasselt University, Hasselt, Belgium; 4 Namur Institute for Complex Systems (naXys) and Department of Mathematics, University of Namur, Namur, Belgium; 5 Artificial Intelligence Lab, Vrije Universiteit Brussel, Brussels, Belgium; 6 Rega Institute for Medical Research, Clinical and Epidemiological Virology, University of Leuven, Leuven, Belgium; 7 Department of Epidemiology and public health, Sciensano, Brussel, Belgium; 8 National Reference Centre for Respiratory Pathogens, University Hospitals Leuven, Leuven, Belgium; 9 Department of Microbiology, Immunology and Transplantation, University of Leuven, Leuven, Belgium; 10 Laboratory of Socioecology and Social Evolution, University of Leuven, Leuven, Belgium; 11 School of Public Health and Community Medicine, The University of New South Wales, Sydney, Australia

**Keywords:** COVID-19, Model, QALY, Vaccine, Non-pharmaceutical intervention, SARS-CoV-2

## Abstract

**Background:**

When formulating and evaluating COVID-19 vaccination strategies, an emphasis has been placed on preventing severe disease that overburdens healthcare systems and leads to mortality. However, more conventional outcomes such as quality-adjusted life years (QALYs) and inequality indicators are warranted as additional information for policymakers.

**Methods:**

We adopted a mathematical transmission model to describe the infectious disease dynamics of SARS-COV-2, including disease mortality and morbidity, and to evaluate (non)pharmaceutical interventions. Therefore, we considered temporal immunity levels, together with the distinct transmissibility of variants of concern (VOCs) and their corresponding vaccine effectiveness. We included both general and age-specific characteristics related to SARS-CoV-2 vaccination. Our scenario study is informed by data from Belgium, focusing on the period from August 2021 until February 2022, when vaccination for children aged 5-11 years was initially not yet licensed and first booster doses were administered to adults. More specifically, we investigated the potential impact of an earlier vaccination programme for children and increased or reduced historical adult booster dose uptake.

**Results:**

Through simulations, we demonstrate that increasing vaccine uptake in children aged 5-11 years in August–September 2021 could have led to reduced disease incidence and ICU occupancy, which was an essential indicator for implementing non-pharmaceutical interventions and maintaining healthcare system functionality. However, an enhanced booster dose regimen for adults from November 2021 onward could have resulted in more substantial cumulative QALY gains, particularly through the prevention of elevated levels of infection and disease incidence associated with the emergence of Omicron VOC. In both scenarios, the need for non-pharmaceutical interventions could have decreased, potentially boosting economic activity and mental well-being.

**Conclusions:**

When calculating the impact of measures to mitigate disease spread in terms of life years lost due to COVID-19 mortality, we highlight the impact of COVID-19 on the health-related quality of life of survivors. Our study underscores that disease-related morbidity could constitute a significant part of the overall health burden. Our quantitative findings depend on the specific setup of the interventions under review, which is open to debate or should be contextualised within future situations.

**Supplementary Information:**

The online version contains supplementary material available at 10.1186/s12889-024-18576-w.

## Background

The SARS-CoV-2 pandemic has been disruptive for the economy and healthcare system and was counteracted by immunisation efforts in 2021–2022 at unprecedented rate and scale in terms of vaccine development as well as distribution, respectively [1]. Despite these efforts, the control of the pandemic was hampered by the emergence of new variants called “Variants of Concern” (VOCs) for which the first generation of vaccines was less effective in reducing transmission [2–5]. Additionally, humoral immunity levels decreased within months after infection or vaccination [6]. Fortunately, protection against severe illness was less impacted by waning of immmunity, although continuous monitoring of the epidemiological situation and additional vaccine uptake efforts were necessary in view of newly emerging VOCs [7].

Mathematical modelling has been widely adopted during the COVID-19 pandemic to inform decision-making by estimating, for example, consequences of unmitigated spread in the initial phase of the pandemic and by quantifying the impact of non-pharmaceutical interventions (NPIs) [8–12]. Even before the deployment of SARS-CoV-2 vaccines, deterministic transmission models were used to study the impact of epidemic response strategies being either mass vaccination or age-targeted vaccination strategies when vaccine supply would be limited [13, 14]. A systematic review concluded that for countries aiming to minimise fatal outcomes, prioritising vaccination of older adults (+65 years) was the optimal strategy, while for countries seeking to minimise infections, prioritising young adults (19-40 years) should be preferred [14]. There were several exceptions to this main conclusion and the findings were sensitive to the identification of high-risk groups with respect to both transmission and severe disease outcome. Hence, targeted modelling studies on vaccination prioritisation are endorsed when country-specific differences in herd immunity profiles and VOC circulation must be considered. Capturing country-specific transmission dynamics is a vital first step in public health planning, as this is determined by both the nature of the pathogen and the network of human contacts through which the disease can spread, which is itself dependent on population age structure and household composition [15].

The introduction of the different VOCs has been time- and country-dependent. The original SARS-CoV-2 strain was followed by the Alpha variant, which increased hospital admissions, ICU load and mortality [16, 17] and started to spread at the end of 2020. The Delta variant emerged in early 2021 with increased severity of illness compared to previous variants [2]. On 26 November 2021, the emerging Omicron strain (B.A.1) was designated as VOC by the World Health Organisation. The growth advantage of the Omicron VOC, due to the improved immune escape and transmission capacity, led to a rapid spread throughout the world, although this VOC demonstrated a reduced risk of clinical severity compared to previous VOCs [18]. However, the intrinsic virulence of Omicron was difficult to estimate given the preexisting immunity in affected populations and the overloaded healthcare system [19]. Subsequently, an Omicron B.A.2 variant emerged in early 2022, although the pathogenicity seemed similar to that of B.A.1 [18].

Vaccines against COVID-19 have been developed for the original SARS-CoV-2 strain and two-dose vaccine schedules showed high effectiveness against symptomatic disease and even higher against severe disease and fatal outcomes [3]. With the Delta VOC, modest reductions in vaccine effectiveness against infection and mild disease were observed, although protection against severe disease remained high for at least 6 months after primary immunisation with two vaccine doses [3]. Booster doses provided a rapid and substantial increase in protection against mild and severe disease [3]. The emergence of the Omicron VOC coincided in several countries with the first booster dose campaign for the elderly, although the rapid rise of the highly infectious and immune-escaping Omicron variant pushed policy makers to accelerate the administration of booster shots to the general population [19].

Several modelling efforts during the COVID-19 pandemic focused on safeguarding healthcare capacity and minimising disease-related mortality [20]. While the life years lost due to COVID-19 mortality are important for quantifying the impact of the measures taken to mitigate the effects of the pandemic, COVID-19 also affected the health-related quality of life of survivors [21], which has often been neglected in previous analyses due to the absence of well-designed data collection. In the end, disease-related morbidity can add up to a large part of the total health burden. To this end, quality-adjusted-life-years (QALYs) provide a quantification of health outcomes in which years of life gained are weighted by the quality of those years. QALYs are widely accepted as a means of informing resource allocation in healthcare, providing a composite measure of health-related benefits [22–26].

In this work, we adapted a previously published stochastic compartmental model to account for the emergence of VOCs and to accommodate the evaluation of COVID-19 vaccination schedules. We performed retrospective scenario analyses for Belgium, accounting for a study population of 11.5 million individuals and focusing on the period August 2021 until February 2022. We integrated data on the age-specific daily incidence of hospitalisations and deaths, disease severity, serial serology, social contacts, and information regarding hospital admission and discharge. We focus on the vaccine uptake trade-off from 2021 between an increase of the first booster dose uptake in adults versus a two-dose vaccine uptake in children. This period was one of the moments in the pandemic with much discussion on vaccine uptake for which also factors not related to epidemiology were at the centre of the debate. As an additional counterfactual scenario, we explored the impact of the emerging Omicron VOC on the preferred strategy. For each intervention strategy, the effect on the burden of disease is evaluated in clinical cases, such as the number of hospital admissions, and in terms of QALYs gained. Our retrospective analysis includes data on vaccine effectiveness, duration of protection, the natural history of the VOC, and quality of life, which was unavailable at the time. The goal is to provide information that could be valuable for future public health management, especially to improve the overall quality of life. As such, this analysis provides information to augment sensitivity analysis when urgent decisions are required and complete information is lacking.

## Methods

The purpose of the analyses presented here was to evaluate distinct vaccine uptake strategies in the context of COVID-19. We aim to achieve qualitative results by exploring counterfactual scenarios driven by vaccine uptake. Therefore, we exploited the unfolding of the Belgian COVID-19 crisis with the induction of SARS-COV-2 in February 2020 and the emerging Alpha, Delta, and Omicron (BA.1 and BA.2) VOCs. Each simulation spans the first two years of the COVID-19 pandemic, running from March 2020 to February 2022. It includes age-specific uptake of first, second, and booster doses of adenovirus and mRNA-based vaccines. The uptake scenarios being examined vary between August 2021 and February 2022, which is the period our results primarily focus on.

### Model structure

**Fig. 1:**
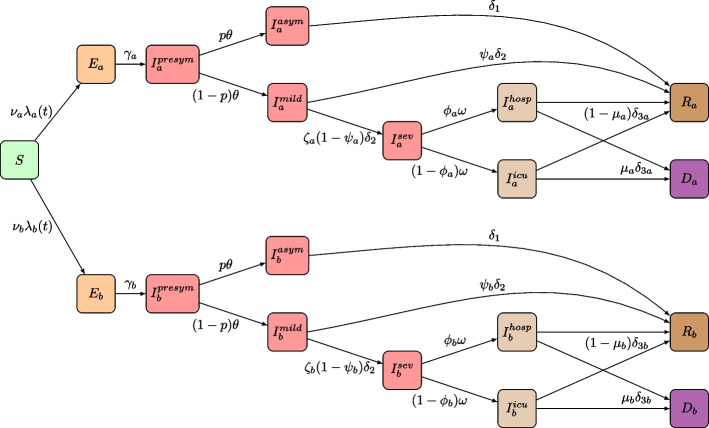
Health states and transitions in the two-strain transmission model. The model structure is described in the main text and model parameters are listed in the Supplementary Information

**Fig. 2:**
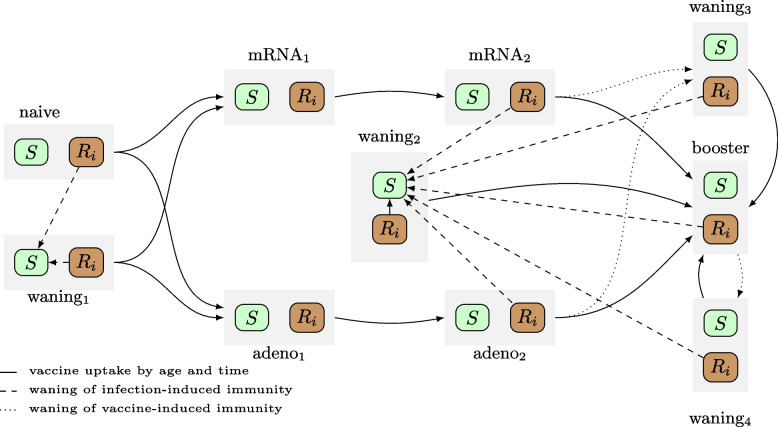
Overview of the duplicated two-strain model structure to account for vaccine type- and dose-specific immunity against infection and severe disease in combination with differential waning immunity over time. The grey boxes embody the transmission structure included in Fig. 1 while only the Susceptible and Recovered are shown here (with $R_i$ representing $R_a$ and $R_b$). More information on the waning states is included in Table 1

We extended a previously published stochastic transmission model for SARS-CoV-2 in Belgium by Abrams et al. [27], by including COVID-19 vaccination, emergence of different VOCs and waning immunity. Our transmission model is a discrete-time age-structured compartmental model with a chain-binomial transition process between various disease compartments that can be categorised into susceptible, exposed, infected, recovered, and death states. Overall, after exposure to the pathogen and acquiring infection, an individual becomes infectious after a latent period and moves to a pre-symptomatic state. Subsequently, individuals develop symptoms or remain asymptomatic, before recovering. Symptomatic infections start mild and have an age-specific probability of progressing to serious illness, implying hospitalisation with or without admission to the ICU. We also account for disease-related mortality of hospitalised cases. The original model formulation is duplicated into a two-strain compartmental structure (see Fig. 1), and transitions between multiple copies of the two-strain model (see Fig. 2) allowed for waning immunity against infection and severe disease. Further elaboration on the construction of the model, specifically based on multiple substructures, is presented in the subsequent paragraphs.

The model structure proposed by Abrams et al. [27] including ten 10-year age groups has been adapted to a two-strain version with a common susceptible class and a duplication of all infection-related health states. Our model structure (see Fig. 1) enabled co-circulation of two variants at the same time with distinct properties with respect to susceptibility, latent period, disease severity, hospital length of stay, mortality, and vaccine-related protection. To cover the newly emerging Delta VOC, we re-used the health states of the dominated original strain in the simulation after book keeping all states. A similar transition was made with the Omicron VOC when the Alpha VOC was fully dominated by the Delta VOC. More information about model dynamics and parameters is provided in the Supplementary Information. Our model operates starting from March 1st, 2020, and accounts for the emergence of new pathogen strains and the administration of various vaccine doses. In the early stages, these factors are represented in small amounts, with heterogeneity and randomness playing critical roles. Even slight variations can become amplified over time. Later on, after COVID-19 vaccination is introduced and attains high coverage, the size of the remaining susceptible population becomes small. This makes the stochastic nature of infection and subsequent processes like hospitalisation and death increasingly significant, especially since the model is calibrated using age-specific incidence data for each of these stages. These elements highlight the importance of the stochastic nature of our compartmental model in accurately reflecting and predicting evolving dynamics.

### Social contact patterns

We used the reported social contact rates of 42 Belgian CoMix survey waves between April 2020 and March 2022 [28, 29] as proxy for effective contacts that allow disease transmission according to the social contact hypothesis [30]. CoMix has been designed as a collection of surveys in which a panel of participants retrospectively reports all social contacts made from 5:00 AM on the day preceding the survey up to 5:00 AM on the day of the survey. A contact was defined as an in-person conversation of at least a few words or a skin-to-skin contact [28]. Changes in transmission that are not directly attributable to changes in contact behaviour are captured in age-specific proportionality factors. They represent, for example, changes in compliance to (social distancing) measures, seasonality effects, and shifts in the location-specific contact intensity (e.g., contacts inside are more risky than contacts outside). For each wave, we estimated age-specific proportionality factors to translate social contact rates into transmission rates that capture age-specific susceptibility and infection-related risk behaviour associated with social contacts [31].

### Variants of concern (VOCs)

The introduction and presence of VOCs in the model population are taken into account in the parameter estimation process based on the baseline genomic surveillance of SARS-CoV-2 in Belgium by the National Reference Laboratory [32]. To simulate the replacement of the original strain in 2021, we aggregated all Alpha, Beta, and Gamma VOC samples that were identified, which we refer to hereafter as Alpha VOC infections, when estimating the penetration of the VOCs into the Belgian population. We attributed the growth advantage of the Alpha VOC completely to transmissibility and ignored the potential effect of immune escape. We assumed that there was no change in the probability of hospital admission for the (aggregated) Alpha VOC. Conflicting post hoc observations have been reported on the severity of this VOC [33]. Therefore, we have chosen to highlight the significant role that increased transmissibility potential plays in hospitalisations and mortality, regardless of any direct effect of the variant on severity.

For the Delta VOC, we account for increased transmissibility and adopted an adjusted hazard ratio for hospitalisation of 2.26 relative to the Alpha VOC based on a cohort study conducted in the UK [34]. Due to the lack of age-specific information to align the reported 95% confidence interval of [1.32;3.89] with our age-specific model design, we opted to use the estimated mean value without considering parameter uncertainty. This adjusted hazard ratio was essential to match the reported incidence of hospitalisations with genomic surveillance data on the Delta VOC [32].

With the emergence of the Omicron VOC, studies [35, 36] indicated a change in the incubation period and the serial interval, which contributes to its transmission advantage. This had a large impact on the estimated reproduction number and the effect of restrictive measures. As such, we included a VOC-specific latent period in our transmission model, which was inferred specifically for the Omicron VOC during the calibration process. Furthermore, Omicron-specific hazard ratios for hospitalisation were pivotal to capture the trends observed in 2022. We adopted age-specific hazard ratios for hospital attendance with the Omicron VOC compared to the Delta VOC, from a cohort study in the UK [37]. More specifically, we used for our 10-year age bands: 1, 0.89, 0.67, 0.57, 0.54, 0.42, 0.32, 0.42, 0.49 and 0.49. The simulation period covers both Omicron sub-lineages BA.1 and BA.2, the latter became dominant in Belgium on February 28th, 2022. The differences in transmission for BA.2 are absorbed in the wave-specific proportionality parameters for February/March 2022.

### COVID-19 vaccines and uptake

All the levels of protection adopted are summarised in Table 1. We used a leaky vaccine implementation approach in which vaccination with 74% effectiveness implies that for a vaccinated individual the probability of acquiring infection is 74% lower compared to a non-vaccinated individual of the same age. Vaccine-induced immunity against infection is implemented as a step function in terms of protection against infection 21 days after the first dose of vaccine. Protection induced by second and booster vaccine doses is assumed to be fully achieved 7 days after vaccine administration (i.e., depending on the maximal effectiveness of the vaccine as reported in Table 1). We consider the differences between mRNA- and adenovirus-based vaccines in how they induce immunity and in terms of protection. We assumed that vaccinated individuals who acquire infection are at a lower risk of hospital admission with COVID-19 and all booster doses in Belgium are mRNA-based vaccines. Given our model structure, reported protection levels against hospital admission were applied as protection against severe disease, which ultimately leads to hospital admission. Vaccinated individuals (with or without a booster) who acquire infection do not have a lower risk of transmitting the disease. This assumption is challenged in the sensitivity analysis.

Vaccine-induced protection and waning immunity have been included through duplication of the two-strain compartmental structure with uptake-based and time-specific transitions (see Fig. 2). This model structure allowed us to explicitly keep track of vaccine type and dose-specific vaccine uptake and to differentiate protection against infection and severe disease between vaccine type and number of doses. The duplicated two-strain compartmental structure also allowed differential waning immunity against infection and severe disease.

We integrated waning immunity into our model by establishing a series of steps transitioning from complete protection to a state of diminishing immunity over an average period of 90 days. In the framework of the compartmental model, the waning rate is defined as the fraction of individuals transitioning from full protection per time unit, which inversely correlates with the average protection duration. Consequently, we incorporated submodels for diminishing vaccine-induced immunity, featuring levels of reduced protection as detailed in Table 1. Initially, infection-induced immunity offers 100% protection, assuming individuals in the “Recovered” state are not susceptible to reinfection. Therefore, our model accounts for the decrease in infection-induced immunity by moving individuals from the “Recovered” to the “Susceptible” compartment within a submodel, which still affords a degree of protection against future infections. We assumed the effect of a booster dose independent of the immunity state upon vaccination, i.e., with or without prior infection or a specific vaccine scheme. We accounted for waning immunity after the booster dose with a dedicated submodel and an average transition time of 90 days. Note that even with waning immunity, vaccinated individuals maintain partial protection against subsequent infection and severe disease upon infection. VOC-specific protection levels for the booster dose have been derived from the literature (see Table 1).

Vaccine uptake in the model is based on age-specific data at the national level reported by the Belgian Scientific Institute for Public Health, Sciensano [38]. By August 2021, on average 90% of the population aged over 20 years had completed their two-dose regimen with mRNA or adenovirus-based vaccines. On the contrary, about 10% of the 0-19-year-olds received two doses of an mRNA vaccine at that time. It is important to note that in August 2021, mRNA vaccines were only authorized for use in children aged 12 years and older by the European Medicine Agency’s Committee for Medicinal Products for Human Use. Subsequently, in 2022, the authorisation was extended to younger children, initially to those aged 6 years and older, and later to infants as young as 6 months of age. The decision to administer booster doses at the end of 2021 was based on the evaluation by the European Medicines Agency that indicated an increase in antibody levels following a booster dose administered about 6 months after the second dose in individuals aged 18 to 55 years. Based on this evidence, first booster doses were recommended in Belgium for people 18 years and older at least 6 months after the second dose.

**Fig. 3:**
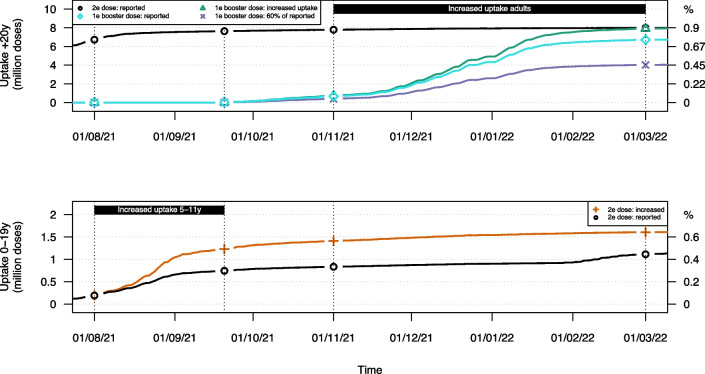
Reported and scenario-based uptake of COVID-19 vaccines over time in adults above the age of 20y (top) and 0-19-year-old children (bottom). The uptake is presented in terms of the absolute number of doses (left axis) and as a percentage of the target group (right axis)

Full details on the type- and dose-specific vaccine uptake by age we included in the model is presented in Fig. S2. We did not explicitly account for risk-group vaccination, since our model structure did not facilitate more subpopulations with differential risk and potentially a more severe episode of COVID-19 disease once infected (i.e., a higher probability of hospitalisation and/or a higher probability of death, if hospitalised). In our analysis, we primarily considered age as the main determinant of risk and severity. The reported uptake of Pfizer-BioNtech (Comirnaty) and Moderna (Spikevax) vaccines are aggregated into one mRNA vaccine type. The relatively low number of reported Johnson & Johnson (Ad26.COV2.S) and Curevac (CV07050101) vaccines were aggregated in the model with the adeno-based AstraZeneca vaccine (ChAdOx1 or Vaxzevria) based on similarities in protection and waning immunity. Third doses (i.e. “first booster dose”) are included in the transmission model as a separate submodel with all health-related compartments. A comprehensive summary of vaccine uptake we included in our model is depicted in Fig. 3, which presents also the scenarios discussed in the subsequent sections of the Methods.

**Table 1:** Vaccine- and infection-induced immunity levels against infection and hospital admissions by VOC and vaccine schedule. The waning-related submodels (e.g. *waning*_*1*_) of Fig. 2 are listed here. The waning rate is the proportion of individuals moving from full protection to reduced protection per unit of time, which is inversely related to the average duration of protection. References and assumptions are provided at the bottom of the table

Vaccine type	Waning	Alpha		Delta		Omicron	
	rate	Infect.^a^	Hosp.^a^	Infect.^b^	Hosp.^c^	Infect.^b^	Hosp.^d^
Adeno: 1st dose	-	49%	76%	43%	76%^e^	18%	65%^f^
Adeno: 2nd dose	-	74%	86%	83%	95%	49%	81%^f^
mRNA: 1st dose	-	48%	83%	72%	79%^e^	32%	65%^e^
mRNA: 2nd dose	-	94%	95%	91%	99%	66%	81%
mRNA: booster dose	-	-	-	95%	99%^g^	67%	90%
Waned immunity after two doses $(waning_3)$	$\frac{1}{90 days}$	63%	92%	63%	92%	9%	57%
Waned immunity after booster dose $(waning_4)$	$\frac{1}{90 days}$	-	-	89%	92%^h^	46%	81%^h^
Waned immunity after infection $(waning_1)$	$\frac{1}{90 days}$	63%^g^	76%^i^	63%^g^	79%^i^	9%^g^	72%^i^
Waned immunity after infection and vaccination $(waning_2)$	$\frac{1}{90 days}$	94%^j^	95%^j^	95%^j^	99%^j^	67%^j^	90%^j^

^a^Bernal et al. [2]; ^b^Andrews et al. [3]; ^c^Andrews et al. [6]; ^d^CDC report,2022; ^e^Assumed equal to 80% of 2 doses; ^f^Assumed equal to mRNA; ^g^Assumed equal after 2 mRNA doses; ^h^Assumed 90% of booster mRNA dose; ^i^Assumed 80% of booster mRNA dose; ^j^Assumed equal after booster mRNA dose

### Parameter estimation

We used Bayesian methods to fit our transmission model to multiple data sources, including daily hospital admissions and bed occupancy, early seroprevalence, genomic surveillance, and mortality data. In order to capture the full extent of the intrinsic variability of the model, we relied on Markov Chain Monte Carlo (MCMC) sampling with 60 chains in the calibration procedure. An adaptive Metropolis-within-Gibbs algorithm was used as MCMC sampler, and parameter priors were based on permutations of previously converged calibration results. The model parameters related to hospital incidence and VOC prevalence were estimated by gradually extending the time horizon over consecutive calibration runs for the stochastic model. The absence of age-specific data on daily hospital discharges and transitions between general wards and ICU hampered a likelihood approach to accommodate hospital occupancy in general and in the ICU. Therefore, the fitting of the model was performed using a multi-step procedure. First, all transmission-related model parameters were estimated while calibrating the model to the observed incidence data on hospitalisation, early seroprevalence and genomic surveillance as described above. Next, all parameters related to hospital and ICU occupancy (including discharge rates) were estimated based on minimising a least squares criterion for the distance between the observed and generated loads. Finally, the estimated mortality-related parameters are inferred again using a likelihood-based approach, distinguishing whether a hospital discharge was due to mortality or recovery. This multi-step procedure has been performed multiple times, of which the final iteration is described in Table S2. Finally, we selected the 40 best performing MCMC chains of the last step to derive parameter estimates for our simulation study.

We used hospital admissions with COVID-19 as a primary source of information to capture the burden of disease. During the development of the model, we observed that around 10%-20% of the admissions with the Alpha and Delta VOCs were primarily due to other pathologies, but patients who tested positive when admitted were transferred to the COVID-19 wards and counted in the COVID-19 hospital load. With the Omicron VOC, the difference between admissions with COVID-19 and for COVID-19 increased even more. Given our focus on hospital capacity, hence occupancy, hospital admissions with COVID-19 were most informative in combination with reported estimates for hospital stay.

We estimated a transmission advantage of the Alpha VOC compared to the original strain of 32% (95% CrI: 24-39%). For the Delta VOC, the transmission advantage compared to the Alpha strain was estimated to be 87% (95% CrI: 71-106%). For Omicron, we estimated an almost instant transition from the exposed to the pre-symptomatic infectious health state (which is in line with the shorter serial interval we referred to previously) and a transmission advantage compared to the Delta VOC of 35% (95% CrI: 9-70%). A comprehensive overview of the model parameters is presented in Table S3 of the Supplementary Information.

The baseline scenario consisted of all estimated parameters during the calibration of the compartmental model and fitted the national trends of SARS-CoV-2 pandemic in Belgium. This includes, for example, the emergence and dominance of the Omicron variant from December 2021 and the observed decrease in hospital admissions and deaths at that time. The full model output from March 2020 is presented in Fig. S4. The transmission model was based on bi-weekly social contact survey data, which allows for including adjusted behaviour over time in the model. The survey data represented changing contact rates, while the estimated proportionality factors captured differences in, for example, contact intensity, susceptibility and infectivity. These factors were age-specific and part of the parameter estimation process (see Fig. S5).

### Quality of life

To estimate the burden of disease, we included the loss of QALYs from a published study on the model-based cost-effectiveness of SARS-CoV-2 vaccination along with physical distancing in the United Kingdom [22]. Disease morbidity estimates were obtained by multiplying the model-based incidence of mild infections, and hospitalised and ICU admitted patients with the QALY loss values in Table 2. Disease-related mortality based on the quality-adjusted life expectancy [39] is obtained by combining the age-specific model estimations on mortality with the Belgian life expectancy for 2019 reported by Statbel [40] and the latest age-specific Belgian population norms based on EQ-5D-5L [25].

**Table 2:** QALY loss of SARS-CoV-2 infection-related health conditions, adopted from Sandmann et al. [22], and the corresponding health states in the transmission model as presented in Fig. 1

Input	Value	Health state	Reference
QALY loss per symptomatic case	0.008	$I_{mild}$	[22, 23]
QALY loss per non-fatal hospitalisation	0.0201	$I_{hosp}$	[22, 24]
QALY loss per non-fatal ICU	0.15	$I_{ICU}$	[22, 26]
QALY loss per fatal infection	Quality-adjusted life years lost	*D*	[25, 40]

### Scenario analyses

We explored retrospective counterfactual scenarios based on vaccine uptake in the presence or absence of the Omicron VOC. None of these scenarios explicitly included the importation of infected cases as a result of international travel except for the introduction of VOCs. We started from the final calibration of the model and the reported vaccine uptake scheme and explored proportionally increased uptake of two doses in 5–11-year-old children and first booster doses in adults over 18-years. Vaccine uptake levels and timing could be explored more in detail with additional objectives and trade-offs, although this analysis aims to provide a basis for predominantly qualitative interpretations. We allow for stochastic variation in the transmission process by running each of the 40 estimated model parameter sets 10 times, hence incorporating 400 model realisations in the final comparison. The number of realisations was determined through a process of model exploration and consideration of the trade-off between model realisations and computational feasibility due to model complexity.

To explore the impact of the uptake of the COVID-19 vaccine, we evaluated an adjustment of the uptake of the first booster dose in adults and an increase in the level of childhood vaccination. First, we changed the uptake of the first booster dose so that it matches the age-specific two-dose uptake levels by 1 March 2022 (see Fig. 3). That is, we assumed that all those eligible for a first booster dose effectively received an mRNA booster dose. The reported first booster dose uptake in the Belgian adult population was 76%, so the additional uptake in the scenario analysis was rather limited. Secondly, we defined a scenario in which we arbitrarily included only 60% of the reported uptake of the first booster dose. The 40% reduction is applied uniformly across all age groups. A third adoption scenario focused on children aged 5-11 years in July-August of 2021. Vaccination in this age class was was not licensed at the time in Belgium, although we explore possible outcomes if 5- to 17-year-old children had been vaccinated simultaneously. More specifically, we aligned the uptake of the mRNA vaccine for children aged 5-9 and 10-11 years with the reported uptake of children aged 12-15 and 16-17 years, respectively. This approach required scaling the reported uptake to match the number of age bins in each group. For example, for each reported first dose in 12-15-year-old children (i.e. 4 age bins), we included $\frac{5}{4}$ dose within the 5-9-year-olds (i.e. 5 age bins) at the given point in time. The time between two consecutive doses is assumed to be three weeks, and the resulting vaccine uptake is presented in Fig. 3. The final uptake of two doses of vaccine is approximately 80% in the group of 10-19 years and 40% in the group of 0-9 years (which corresponds to 80% in the group of 5-9 years).

### Sensitivity and robustness

To assess the impact of the Omicron VOC on the epidemic trajectory, we performed simulations of our three adjusted vaccine uptake scenarios without the presence of the Omicron VOC, while keeping all other model parameters constant.

In our main analysis, we adopted a conservative approach that assumed no infectiousness-related protection from the COVID-19 vaccine, which potentially underestimates the effect of the intervention. As such, we opt to minimise the risk of overestimating the intervention-related benefits for this exploratory analysis. However, household studies conducted in Denmark [41] and the UK [42] have reported a 31%-45% decrease in the risk of SARS-CoV-2 transmission among vaccinated individuals. Therefore, as a sensitivity analysis taking into account these findings, we performed a comprehensive model calibration assuming a 30% reduction in infectiousness for vaccinated individuals and exploring the effect on the vaccine scenarios.

As robustness analyses, we performed the full model calibration with invariant proportionality factors across different consecutive CoMix waves. A single set of age-specific parameters was not possible as the link between observed contact rates and disease transmission was not constant throughout 2020–2022 due to differences in contact intensity, duration, and location, among other things. Therefore, we aggregated the CoMix waves into five groups based on the distancing measures that were in place, the (school) holiday periods, and the model fit. For the time period without CoMix data (i.e. for March and September–November 2020), we still required time-specific q-factors. Details are provided in Supplementary Table S1.

### Study design

Part of this epidemiological mathematical modelling study was carried out to inform the Belgian government and the general public about COVID-19 trends and possible interventions. This study is based on data sources from the Belgian Institute of Public Health (Sciensano) in combination with published estimates and data sets (e.g., CoMix). Funding agencies did not have a role in study design, data collection, data analysis, data interpretation, reporting, or in the writing of this manuscript. Data preparation and statistical analyses were performed using R (version 4.2.2; R Foundation for Statistical Computing, Vienna, Austria) on MacOS 12.5 and using R (version 4.0.2) on Rocky Linux 8.8 on the VSC cluster.

## Results

**Fig. 4:**
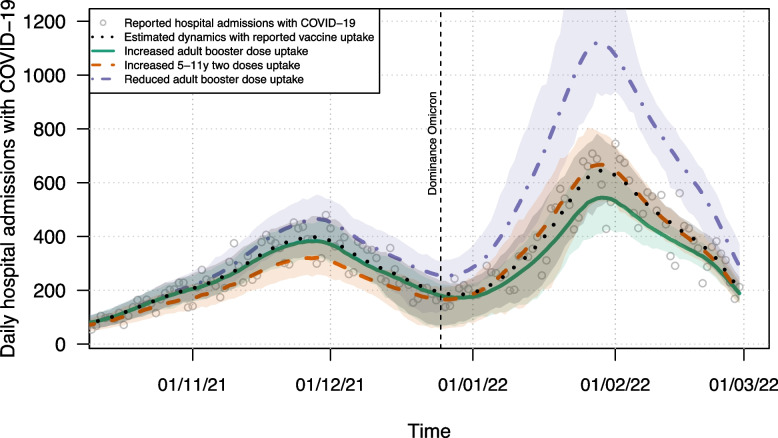
Projected daily hospital admissions with COVID-19 in Belgium with adjusted vaccine uptake for 5-11-year old children or adults. The curves represent the time-dependent point-wise average of 400 stochastic realisations, while the 95% credible interval is shown by the shaded area. Related figures on hospital occupancy, ICU load and mortality are included in the Supplementary Information

We analysed the effects of age-specific COVID-19 vaccination and the emerging Omicron VOC on the burden of disease by building on a stochastic compartmental transmission model developed for Belgium [27]. As such, we explored distinct vaccine allocation policies to explore different scenarios by covering the first two years of the COVID-19 pandemic (i.e., from March 2020 to February 2022). The model accounted for age-specific uptake of first, second and booster vaccine doses of adeno- and mRNA-based COVID-19 vaccines. We fitted the model parameters using the reported vaccine uptake and could reproduce the daily number of new hospitalisations and deaths, and the daily total number of hospitalised and ICU admitted patients in Belgium (see Fig. 4). Early serial serological data and information from the literature on the relative hospital hazard ratio of each VOC were essential to capture the observed dynamics. Subsequently, the model extrapolated the temporal burden of disease in the Belgian population, estimating a total of 8.9 million infections (including re-infections) until February 2022. Table 3 summarises our analysis showing the estimated burden of disease in absolute numbers for different clinical outcomes (i.e., death, ICU and hospital admission, mild infections, total infection), and QALYs, as well as the incremental effects for the different vaccine uptake scenarios.

**Table 3:** Projected burden of disease between March 2020 and February 2022 and the incremental burden of disease for the vaccine uptake scenarios under study. We did not account for adjusted social contact behaviour in the scenarios due to increased/reduced virus circulation and disease morbidity, or to (non-)vaccination. The table presents the reported and incremental vaccine uptake and results are provided in terms of means and corresponding 95% credible interval. Stochastic differences between the baseline and altered vaccine uptake scenario can give rise to the estimation of counterintuitive incremental effects. This model artefact is visible in some of the credible intervals

	Burden of disease from March 2020 until Feb 2022	Incremental results with increased adult booster dose uptake	Incremental results with increased vaccine uptake 5-11y in 2021	Incremental results with reduced adult booster dose uptake
**Deaths**	29,053 [27,817;30,090]	-310 [-616;-2]	-325 [-723;36]	+1,874 [2,522;1,434]
**ICU admissions**	30,544 [29,700;31,243]	-532 [-950;-70]	-703 [-1,350;-33]	+3,098 [4,066;2,440]
**Hospital admissions**	139,506 [135,241;142,680]	-4,019 [-7,068;-866]	-3,171 [-8,101;364]	+20,035 [27,406;15,048]
**Mild infections**	4,577,231 [4,201,783;5,008,672]	-217,773 [-416,007;-48,904]	-50,969 [-365,957;145,356]	+638,486 [1,031,606;381,605]
**Total Infections**	8,906,170 [8,196,824;9,745,087]	-418,233 [-806,964;-86,812]	-116,518 [-736,416;269,100]	+1,173,796 [1,916,998;683,533]
**QALYs lost**	259,687 [250,019;266,080]	-4,512 [-8,505;-361]	-3,809 [-10,717;1,341]	+21,057 [15,225;31,582]
**Vaccine Uptake (doses)**	25,671,309	+1,227,329	+905,113	-2,896,104
$\frac{\mathbf {QALY\ gain\ (mean)}}{\mathbf {Uptake\ (doses)}}$	-	0.0037	0.0042	0.0054

### Hospital admissions

With a first booster dose uptake level in adults equal to the coverage of the primary two-dose COVID-19 vaccination, we observed a reduction in hospital admissions compared to the baseline scenario (green versus black curves, respectively, in Fig. 4). Note that more than 80% of the +50-year-old population most at risk of severe infection and hospitalisation with COVID-19 already received their first booster dose, which partly explains the limited effect of additional booster dose uptake. On the contrary, if we reduced the first booster dose uptake to 60% of the reported uptake, the model projected higher levels of morbidity and mortality for the study period (purple curve in Fig. 4). Specifically, the projected maximum values for hospital admissions and load were doubled compared to the respective values in the baseline scenario.

The projected disease burden while considering a scenario of increased vaccination uptake for children aged 5-11 years (orange curve in Fig. 4) showed divergent results in terms of the relative ranking of the estimated burden. As such, we observed a relative reduction in hospitalisations in 2021, when the Delta VOC was circulating, but a relative increase in January-March 2022 following the emergence of Omicron. However, in terms of ICU load, the projected peak occupancy was lower in the case of increased childhood vaccination (mean 723 with 95% Credible Interval (CrI) [588;858]) compared to the increased adult booster dose uptake (842 [702;991]).

### QALYs and age-specific burden

**Fig. 5:**
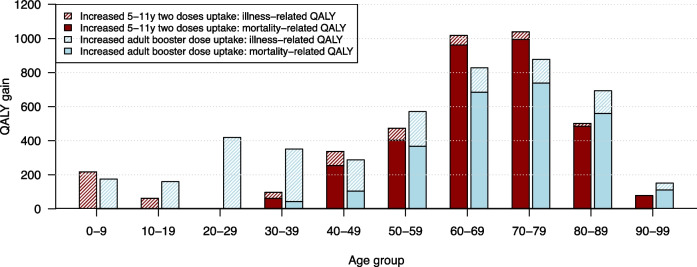
Average age-specific QALY gain with the increased childhood vaccination in Jul-Sept 2021 (red, left) and the increased first booster dose uptake in adults by Feb 2022 (blue, right). The effects are presented by illness-related, i.e. due to mild and severe infections, and mortality-related QALY gain

We estimated an average utility loss for Belgium due to COVID-19 at 259,687 QALYs as a result of disease-related mortality and morbidity from March 2020 up to February 2022. Scenario analysis showed that increased uptake of adult booster doses could have gained on average 4,512 QALYs (Table 3). This could be translated in the expected healthy life years of 65 newborns, based on the estimated quality-adjusted life expectancy for the UK of 70 years [39]. The estimated gain in QALYs associated with increased vaccine uptake in children aged 5-11 years was lower, but it did induce a reduction with respect to mortality and ICU admissions. The counterfactual scenario with reduced uptake of first booster doses shows an average loss of 21,057 QALYs. Note that none of the simulations account for adjusted behaviour or NPIs when the epidemic situation improved or deteriorated, or for potential changes in contact behaviour due to vaccination [43]. We also compared the age distribution of health-related benefits for scenarios with increased uptake (Fig. 5). When increasing the uptake of the first booster dose in adults, we observed more illness-related QALY gains in general and especially in the active population (20-59 years). Increased vaccine uptake for children aged 5-11 years demonstrated greater mortality-related QALY gains for the 60-79-year-old population, that is, due to reduced overall transmission levels.

The QALY gain observed for 0-9-year-olds, as shown in Fig. 5, is attributed to prevention of disease morbidity. Due to the utilisation of 10-year age groups in our simulation, we were unable to provide a more detailed breakdown for the 5-11-year age range. However, our simulation revealed that the increased uptake of vaccines in 5-11-year-olds resulted in an average prevention of 62,500 infections within the category of 0-9-year-old age (Table S5). This corresponds on average to approximately 23,700 mild infections and 1,250 hospital admissions for this young age group.

### Omicron VOC

**Fig. 6:**
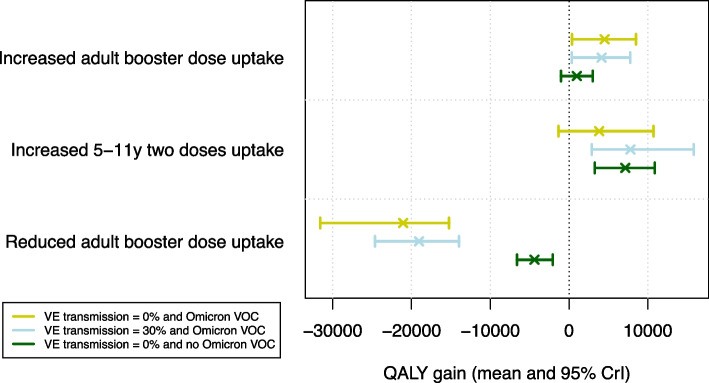
Sensitivity analysis on the projected QALY gain when assuming 0% or 30% vaccine-effectiveness against transmission and in the absence of the Omicron VOC. Results are shown per vaccine uptake scenario in terms of the mean (cross) and corresponding 95% CrI (whiskers)

The emergence of the Omicron VOC resulted in adjustments in terms of NPIs in several countries. As such, it is likely that it also affected the preferred vaccine uptake strategy. To explore this effect, we performed scenario analyses omitting the emergence of the Omicron VOC, which showed a significant impact on the QALY gain (see Fig. 6). All projections showed a decrease in morbidity and mortality with almost no burden by February 2022. When comparing the uptake scenarios under study in the absence of Omicron, additional vaccination uptake in children was shown to be more effective than the increased first booster dose uptake in adults. The projected hospital admissions and ICU occupancy were lower and we estimated an incremental QALY gain of 7,135 (95% CrI: [3266;10,881]). Increasing the uptake of first booster doses in adults showed limited impact in terms of QALY gain in the absence of Omicron VOC.

### Sensitivity analyses

By default, we conservatively assumed no vaccine-related protection in terms of infectivity, therefore a breakthrough infection is equally infectious as an infection in the absence of vaccination. However, we account for indirect vaccine-related protection in terms of transmission, since when an infection is prevented, subsequent transmission is also prevented. To analyse the sensitivity of our results to infectiousness-related protection, we performed the calibration of the model assuming vaccine-related protection against transmission of 30%. The re-calibrated model also captured the reported burden of disease in terms of hospital admissions, prevalence of VOCs, and mortality, although the scenario analysis produced different results (see Fig. 6). In particular, when assuming infectiousness-related vaccine protection, the scenario in which vaccination uptake increased among 5- to 11-year-olds in 2021 resulted in a greater average gain in QALY compared to the scenario of increased first booster dose uptake in adults.

For each set of social contact data from the, on average, bi-weekly CoMix survey, we estimated age-specific proportionality factors to translate social contact rates into transmission rates that capture age-specific susceptibility and infection-related risk behaviour. To assess the sensitivity of our scenario results for the numerous age-specific factors, we performed an additional full calibration using proportionality factors across consecutive CoMix waves. Therefore, the waves were grouped according to the presence of distancing measures, (school) holiday periods, and model fit evaluation. Limited changes in age-dependent proportionality factors allowed temporal variations in factors such as contact location and intensity, while we relied on CoMix data for behavioural changes related to contact rates and age preferences. The projections of the resulting model with the reduced set of proportionality parameters also matched the reported incidence (see Fig. S10), although less precise. The corresponding scenario analysis with age-specific vaccine uptake programmes showed similar results in terms of the prevented burden of the disease.

## Discussion

In this work, we explored two distinct COVID-19 vaccine programmes: increasing two-dose uptake in children versus increasing the uptake of the first booster dose in adults. While the average gain in QALYs was higher for the scenario with increased booster dose uptake in adults, the distribution of health benefits by age and the discrepancy between QALY gains achieved through reduced mortality and those achieved through reduced morbidity warrant thorough evaluations. Prevention of severe disease resulting in incapacitated health care delivery (mainly in hospitals), and of mortality was prioritised when designing initial COVID-19 vaccination strategies. Many health economists and ethicists, including Giubilini et al. [44] drew attention to more traditional outcomes like QALYs, and inequality indicators, such as the age distribution of QALY gains as a source of complementary information for policy makers. Prioritisation based on any outcome necessitates evaluating the (cost-)effectiveness of different vaccines in achieving these outcomes, based on how well they reduce infection, disease and death in different target groups.

Infectious disease prevention has many additional societal benefits, most obviously a reduction of illness-related absenteeism and productivity losses [45, 46]. For COVID-19, Maltezou et al. [47] showed that primary mRNA vaccines prevented approximately seven out of ten absenteeism episodes among hospital-based healthcare workers. Another hospital study in Greece, during the Delta and Omicron waves, showed that unvaccinated COVID-19 patients had significantly longer absence periods compared to vaccinated COVID-19 patients [48]. Our scenario with increased uptake of the first booster dose in adults showed a reduction of 217,000 mild infections, constituting a major economic impact. In addition, preventing infections in children has broader implications beyond reducing disease burden, notably improving school attendance and mitigating the associated burden and opportunity losses for their families. As such, it is a limitation from a societal viewpoint that these prevented costs of absence are ignored in this analysis.

A cost-utility model for Brazil based on a decision tree model showed that each Vaxzevria adeno-based vaccine had an incremental effectiveness of 0.002 QALYs over the course of one year [49]. This magnitude aligns with our findings (see Table 3) although our estimates are higher due to the inclusion of mRNA vaccines and the adoption of a dynamic model. Mathematical transmission models enable capturing infectious disease dynamics throughout the pandemic in which infections do not occur uniformly over time. As such, the expected benefit with a dynamic model is higher since it accommodates herd immunity, i.e. the reduction of secondary cases.

The emerging VOCs have had an important impact on the course of the pandemic. The modelling study by Sonabend et al. [50] found that the Delta VOC interfered with decision making on pandemic control measures in the UK. They concluded that with the Delta variant, it was not possible to fully lift NPIs in September 2021 without causing a third wave of hospital admissions and deaths, even if vaccination coverage was high. As such, the transmissibility of VOCs and the corresponding vaccine effectiveness must be carefully monitored when countries relax pandemic control measures [51]. In our analysis, we showed that the Omicron VOC interfered with the preferred vaccine intervention for Belgium. Without the Omicron VOC, increasing uptake in 5–11-year-old children during the summer of 2021 in our simulation study was most rewarding in terms of QALYs. With the Omicron VOC, increasing uptake of the first booster dose in adults in the fall of 2021 was most rewarding in terms of QALYs.

The scenario involving increased vaccine uptake in children aged 5-11 years contributes to the multifaceted debate surrounding COVID-19 vaccination in this age group and the associated risk of myopericarditis [52, 53]. However, SARS-CoV-2 infections also increased the risk of myopericarditis [54, 55], highlighting the importance of preventing infections to mitigate the risk of myopericarditis. Given that most of the available information focused primarily on adults or 12-17-year-olds, it was not feasible to estimate a credible net balance for 5-11-year-olds in terms of the expected risk of Myopericarditis associated with our scenarios. However, we do observe a direct positive effect of the vaccine uptake scenario for this age group in our simulations, in addition to its indirect impact on the overall population.

Mathematical models are suited for scenario analyses to investigate future or retrospective counterfactual trajectories. Previous work, based on a combined-cohort Markov decision tree model for the US, showed that booster doses of the bivalent Pfizer-BioNTech COVID-19 vaccine generates notable public health gains [56]. We made a deliberate decision not to exclude all booster doses as a counterfactual scenario, in order to assess the additional benefits provided by the uptake of the first booster dose. When scenario analyses are performed with large alterations regarding vaccine uptake, the inherent assumption that social contact behaviour and NPIs do not change becomes uncertain when the scenario results in an excessive or very limited disease burden. On the one hand, an excessive burden of disease could lead to collapsed healthcare systems and even more fatal outcomes, or voluntary behavioural changes due to prevalence, awareness and perception of the disease, which have a controlling effect. On the other hand, when the disease prevalence is very low, there may be a decrease in the adherence to NPIs. Consequently, the projected impact of extremely different vaccine uptake may not be fully realised, raising doubts about the validity of the results obtained from such scenarios. While our study uses hypothetical uptake scenarios, we grounded our increased uptake levels in observed dynamics to ensure feasibility. For instance, we assumed that vaccine acceptance among 5-11-year-olds would reflect that observed in 12-15-year-old children, or that all adults who received two doses would be willing to receive a first booster dose.

Serial serological data collected in the initial phase of the pandemic has been most informative to model the first COVID-19 wave in Belgium [11, 27, 57]. Despite its availability early on, initial serological data collections have been discontinued. However, serial serology across the entire period under study would be valuable, especially for disentangling differences in transmission dynamics from changes in severity induced by emerging VOCs when studying, for example, the incidence of reported hospital admissions. Information from the literature on the relative hospital hazard ratio for each VOC was used as an alternative to describe the observed dynamics. A secondary goal of this analysis was to estimate the total number of infections in the population, since clinical testing is affected by symptom prevalence, availability, adherence, and testing policy, which changed at several occasions in Belgium between 2020 and 2022.

The time-varying proportionality factors allowed to correct for transmission-related elements that are not captured by changes in intensity and structure of the collected social contact data, such as ventilation, but also have been useful in monitoring the structure and assumptions of the model. When, for example, the estimated factors increased rapidly for subsequent waves, we noticed that susceptibles were depleted in an unrealistically fast way to be able to reproduce the reported number of hospital admissions. As such, we had to, among others, include waning immunity or VOC-related parameters in terms of the hospital hazard ratio for the Delta VOC and the serial interval for the Omicron VOC. After doing so, the proportionality factors stabilised, while the model results still matched the reported incidence.

Any model is a simplification of reality and, therefore, depends on the assumptions made. Our compartmental model does not account for local differences in immunity and clustered social contact next to age groups. General trends are well captured, although local outbreaks are underestimated and herd immunity effects are overestimated in subpopulations with immunity levels below the national level. This could be resolved by relying on metapopulation [58] or individual-based modelling [59, 60] tools that provide high-resolution mechanistic explanations of transmission dynamics that are relevant in pandemic response planning, potentially at the cost of an increased computational burden.

The compartmental model that we adopted, which features a large number of age groups, different viral strains and vaccine-related characteristics, is inherently complex. Our model choice is supported by the nature of our reference data and scenarios, which are orientated towards the population level. Given the extensive number of compartments, an individual-based model might seem suitable, offering potentially clearer and more interpretable rules and logic [61]. However, individual-based models come with significant computational demands and the challenge of parameter inference, necessitating many realisations of the underlying simulation model. To investigate strategies on a finer scale, by e.g. including household transmission dynamics, an individual-based model could be more appropriate.

We rely on scenario analyses to estimate relative changes, although further sensitivity analyses are warranted and the quantitative results should be interpreted with caution. We chose a uniform approach (i.e., in which a decrease in vaccine uptake is uniformly imposed across all age groups) to model the reduced uptake of booster dose in all age groups, serving as a conservative reference. The impact of reduced booster uptake can be higher or lower, depending on the relative reduction among the elderly compared to other age groups. The evaluation of different age-specific reductions was omitted to maintain a minimal and focused set of scenarios for comparison. While we employed a lifetime horizon to capture potential long-term benefits, we did not include long COVID in our study due to the complexities in estimating its incidence, which is influenced by factors like age, infection and vaccination history, and disease severity. In particular, the uncertain vaccine effectiveness against long COVID prompted us to exclude this aspect from our analysis. This exclusion likely led to an underestimation of the burden and, consequently, the potential benefits of prevention strategies. In terms of feasibility and data availability in 2021, we assumed that the generation interval from 2020 [62] remained constant up to the Delta VOC. This has been questioned by Hart et al. [63], therefore, we could overestimate the advantage of Delta with respect to infectiousness and susceptibility. With the emergence of the Omicron VOC, an adjustment in terms of the average generation interval was indispensable to capture the reported burden of disease. As such, assumptions related to the generation interval (distribution and its mean) have been important in investigating the dynamics and control of new variants of SARS-CoV-2, which is also found in other studies [64, 65].

## Conclusions

Our findings highlight general and age-specific outcomes associated with SARS-CoV-2 vaccination. Increasing vaccine uptake in children aged 5-11 years showed a lower incidence and occupancy of the ICU, which has been a key indicator for the implementation of NPIs and the preservation of the optimal functioning of the healthcare system. If we assume that the vaccine also has a direct effect on infectiousness, the impact of the childhood vaccination programme increases substantially. However, this could be countered by a reduced vaccine effectiveness against infection for children [66]. In any case, both scenarios studied could have required less NPIs, which in turn could have led to improvements in economic activity and mental well-being [67]. The COVID-19 pandemic has had differential effects on various populations, particularly those who are economically disadvantaged or have preexisting health conditions, emphasising the interconnected nature of inequalities [68]. In the context of vaccine strategies, it is vital to recognise equity in accessing healthcare services, ensuring fairness and social justice in the distribution of vaccines.

### Supplementary Information

**Supplementary Material 1.**

## Data Availability

The series of COVID-19 cases and hospital admissions were publicly available from the Belgian Institute of Public Health (Sciensano). Data on VOC have been obtained from the Belgian SARS-CoV-2 sequencing consortium, coordinated by the national reference centre UZ/KU Leuven. Longitutinal data on admissions with COVID, mortality, and age distribution of hospital cases have been obtained from Sciensano. All input data and model code required to perform the published scenarios from our GitHub repository are available in a public archive on ZENODO [69].

## Abbreviations

COVID-19: Coronavirus disease 2019
QALY: Quality-adjusted life year
VOC: Variants of Concern
SARS-CoV-2: Severe acute respiratory syndrome coronavirus 2
ICU: Intensive care unit
NPI: Non-pharmaceutical interventions
mRNA: messenger ribonucleic acid
CoMix: longitudinal, multi-country social contact survey
CrI: Credible interval
