# Supplementary material for: The impact of quality-adjusted life years on evaluating COVID-19 mitigation strategies: lessons from age-specific vaccination roll-out and variants of concern in Belgium (2020-2022)
Source: BMC Public Health. 2024 Apr 26;24:1171. doi: 10.1186/s12889-024-18576-w (PMC11047051; doi:10.1186/s12889-024-18576-w)
Supplement: Supplementary file 1 — Supplementary Material 1. [file 12889_2024_18576_MOESM1_ESM.pdf]

Supplementary Information: The impact of quality-adjusted life years on evaluating COVID-19 mitigation strategies: lessons from age-specific vaccination roll-out and variants of concern in Belgium (2020-2022)

Contents

S1 Compartmental model structure . . . 2
- S1.1 Force of infection . . . 3
- S1.2 Social contact rates . . . 4
- S1.3 Immunity . . . 6
- S1.4 Discrete time stochastic epidemic model . . . 7

S2 Vaccine uptake . . . 10

S3 Model calibration . . . 12
- S3.1 Model initialisation . . . 13
- S3.2 Estimation . . . 14
- S3.3 Overview of model parameters . . . 15

S4 Estimated burden of disease . . . 17

S5 Proportionality factors . . . 18

S6 Additional results . . . 19

S7 References (Supplementary Information) . . . 22

S1 Compartmental model structure

The transmission model is a discrete-time stochastic compartmental chain-binomial model designed to describe COVID-19 and is based on the methodology published by Abrams et al. [1]. The structure of the two-strain model including susceptible, exposed, infectious, recovered and deceased health states is presented in Figure S1, which is a duplication of Figure 1 shown in the main text. Individuals in the red compartments are able to transmit the disease. Individuals are susceptible to infection when in compartment $\boldsymbol{S}$ (with boldface notation representing a vector that includes age-specific rates), and after effective contact (between a susceptible and an infectious individual for variant a or b) the susceptible individual moves to an exposed state $\boldsymbol{E_a}$ or $\boldsymbol{E_b}$, respectively. The transition from the susceptible to exposed compartments is based on an age-, time- and variant-specific rate $\boldsymbol{\lambda}_i(t)$, called the force of infection. The subscript i $(i = a, b)$ refers to the specific variant in the two-strain model structure and will be used throughout the following description thereof. The force of infection (i.e., $\boldsymbol{\lambda_i(t)}$) can be reduced by vaccine-induced immunity against infection denoted by ν_i.

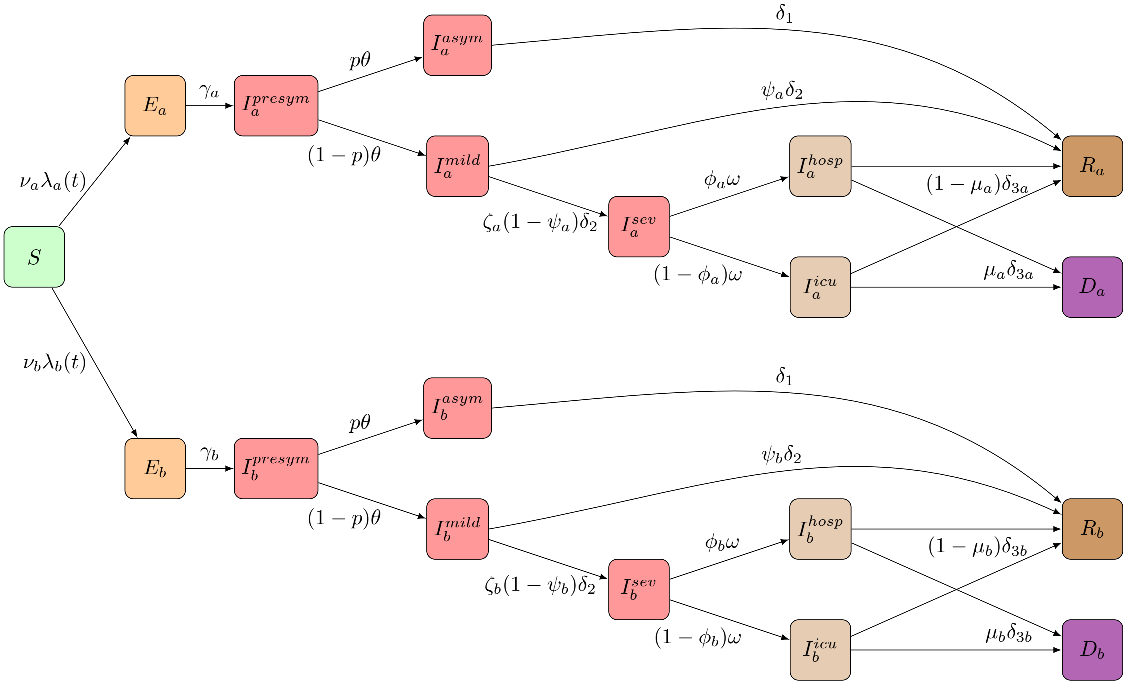

Figure S1: Schematic overview of the health states in the compartmental two-strain transmission model.

After a latent period in state $\boldsymbol{E_i}$, the individual becomes infectious and moves to a pre-symptomatic state $\boldsymbol{I_i^{presym}}$ at rate γ_i. Subsequently, individuals develop symptoms (state $\boldsymbol{I_i^{mild}}$) with probability $1-\boldsymbol{p}$ or remain completely free of symptoms in compartment $\boldsymbol{I_i^{asym}}$ with probability $\boldsymbol{p}$). Asymptomatic cases recover at a rate δ_1. Symptomatic infections are very mild and such cases recover at rate δ_2 with probability $\boldsymbol{\psi_i}$ or move to a state $\boldsymbol{I_i^{sev}}$ prior to hospital admission at rate δ_2 with probability $(1 - \boldsymbol{\psi_i})$. The latter can be reduced by vaccine-induced immunity against severe disease ζ_i. When being seriously ill, implying hospitalisation,

people move with rate $\boldsymbol{\omega}$ to state $\boldsymbol{I_i^{hosp}}$ and probability $\boldsymbol{\phi}_i$ or become critically ill ($\boldsymbol{I_i^{ICU}}$) with probability $(1 - \boldsymbol{\phi}_i)$. Hospitalised and critically ill patients admitted to the Intensive Care Unit (ICU) leave the hospital at a rate δ_{3i} with an age-specific case fatality probability $\boldsymbol{\mu_i}$ (that is, the likelihood of dying when severely ill and hospitalised).

The adopted model structure enables the co-circulation of two VOCs while accounting for a VOC-specific latency period (γ_i^{-1}), probability of hospitalisation $(1 - \boldsymbol{\psi_i})$, probability of ICU admission when hospitalised (1-$\boldsymbol{\phi}_i$), hospital length of stay δ_{3i}^{-1} and case-fatality probability ($\boldsymbol{\mu_i}$). Vaccine related parameters, ν_i and ζ_i are discussed in Section S1.3.

The following set of ordinary differential equations describes the (deterministic version of the) flows in the proposed age-structured two-strain compartmental model presented in Figure S1 with i representing strain a and b:

$$\begin{aligned}
\frac{d\boldsymbol{S}(t)}{dt} &= -\nu_a \boldsymbol{\lambda_a(t)S(t)} - \nu_b \boldsymbol{\lambda_b(t)S(t)} \\
\frac{d\boldsymbol{E_i}(t)}{dt} &= \nu_i \boldsymbol{\lambda_i}(t)\boldsymbol{S(t)} - \gamma_i \boldsymbol{E_i(t)} \\
\frac{d\boldsymbol{I_i^{presym}(t)}}{dt} &= \gamma_i \boldsymbol{E_i(t)} - \theta \boldsymbol{I_i^{presym}(t)} \\
\frac{d\boldsymbol{I_i^{asym}(t)}}{dt} &= \boldsymbol{p}\theta \boldsymbol{I_i^{presym}(t)} - \delta_1 \boldsymbol{I_i^{asym}(t)} \\
\frac{d\boldsymbol{I_i^{mild}(t)}}{dt} &= (1 - \boldsymbol{p})\theta \boldsymbol{I_i^{presym}(t)} - \{\boldsymbol{\psi_i} + \zeta_i(1 - \boldsymbol{\psi_i})\delta_2\} \boldsymbol{I_i^{mild}(t)} \\
\frac{d\boldsymbol{I_i^{sev}(t)}}{dt} &= \zeta_i(1 - \boldsymbol{\psi_i})\boldsymbol{I_i^{mild}(t)} - \boldsymbol{\omega I_i^{sev}(t)} \\
\frac{d\boldsymbol{I_i^{hosp}(t)}}{dt} &= \boldsymbol{\phi_i \omega I_i^{sev}(t)} - \delta_{i3} \boldsymbol{I_i^{hosp}(t)} \\
\frac{d\boldsymbol{I_i^{icu}(t)}}{dt} &= (1 - \boldsymbol{\phi_i})\boldsymbol{\omega I_i^{sev}(t)} - \delta_{i3} \boldsymbol{I_i^{icu}(t)} \\
\frac{d\boldsymbol{D_i}(t)}{dt} &= \boldsymbol{\mu_i}\delta_{i3} \left\{ \boldsymbol{I_i^{hosp}(t)} + \boldsymbol{I_i^{icu}(t)} \right\} \\
\frac{d\boldsymbol{R_i}(t)}{dt} &= (1 - \boldsymbol{\mu_i})\delta_{i3} \left\{ \boldsymbol{I_i^{hosp}(t)} + \boldsymbol{I_i^{icu}(t)} \right\}
\end{aligned}$$

where, for example, $\boldsymbol{S} = (S_1(t), S_2(t), \ldots, S_K(t))^T$ represents the vector of the number of susceptible individuals in age group $k = 1, \ldots, K$ in the population at time t. The age-structured compartmental transmission model consists of 10 age classes, i.e., [0-10), [10-20), [20-30), [30-40), [40-50), [50-60), [60-70), [70-80), [80-90), [90, ∞) with the number of individuals at the start of the pandemic in each age class obtained from Eurostat.

S1.1 Force of infection

The VOC-specific force of infection is calculated as follows (with $i = a, b$ representing the two VOCs under consideration and represented in the model at time t):

$$\boldsymbol{\lambda_i(t)} = \tau_i \boldsymbol{\beta^{sym}(t)} \left\{ \boldsymbol{I_i^{mild}(t)} + \boldsymbol{I_i^{severe}(t)} \right\} + \tau_i \boldsymbol{\beta^{asym}(t)} \left\{ \boldsymbol{I_i^{presym}(t)} + \boldsymbol{I_i^{asym}(t)} \right\},$$

with τ_i a VOC-specific scaling factor related to the transmission potential relative to the original strain, and the age-, time- and symptom-specific transmission parameters

$$\boldsymbol{\beta^{sym}(t)} = \overline{q}\boldsymbol{q(t)C^{sym}(t)}$$
$$\boldsymbol{\beta^{asym}(t)} = r\overline{q}\boldsymbol{q(t)C^{asym}(t)},$$

with $\overline{q}$ a single proportionality factor estimated for March 2020, $\boldsymbol{q(t)}$ the time- and age-specific proportionality adjustment factor, $\boldsymbol{C^{sym}(t)}$ the age- and time-specific contact rates per capita for symptomatic individuals, $\boldsymbol{C^{asym}(t)}$ the corresponding contact rates per capita for healthy and asymptomatic individuals and r the relative infectiousness of pre-symptomatic and asymptomatic individuals as compared to infectiousness for symptomatic cases.

S1.2 Social contact rates

Age- and time-specific contact rates per capita for healthy and asymptomatic individuals ($\boldsymbol{C^{asym}(t)}$) are based on the Belgian longitudinal social contact survey CoMix [2]. The reported social contact rates of 43 survey waves between April 2020 and March 2022 were aggregated into a contact matrix per survey wave and scaled to match the contact rate, or average number of contacts per day, for one susceptible individual with one infected individual in the population. As such, the model adopted 43 different social contact patterns, each represented by a 10×10 social contact matrix (with both symptomatic and asymptomatic contact matrix versions as discussed hereunder).

We assumed that individuals do not change their contact behaviour when being asymptomatically infected with SARS-CoV-2. The overall contact matrix is the sum of contact matrices encompassing contacts made at the following locations: home, work, school, transportation, leisure, and other places. Thus $\boldsymbol{C}^{asym}$ is obtained as follows:

$$\boldsymbol{C}^{asym} = \boldsymbol{C}^{home} + \boldsymbol{C}^{work} + \boldsymbol{C}^{school} + \boldsymbol{C}^{leisure} + \boldsymbol{C}^{transport} + \boldsymbol{C}^{other}$$

The contact matrices for symptomatic individuals are obtained by rescaling the matrix $\boldsymbol{C}^{asym}$ at the respective locations by the relative change in the number of contacts as quantified by Van Kerckhove et al. [3] during the 2009 A (H1N1) Influenza pandemic in England. Hence, we presume that social contacts are adapted in a similar way in the Belgian population upon contracting the disease and experiencing symptoms. Thus, $\boldsymbol{C}^{sym}$ is defined as a weighted sum of the aforementioned contact matrices at specific locations, i.e.,

$$\boldsymbol{C^{sym}} = \boldsymbol{C^{home}} + 0.09\boldsymbol{C^{work}} + 0.09\boldsymbol{C^{school}} + 0.13\boldsymbol{C^{transport}} + 0.06\boldsymbol{C^{leisure}} + 0.25\boldsymbol{C^{other}}$$

To account for the temporal transition in social contact behaviour between cross-sectional time points of measuring social contact rates, we included a linear change over a 6-day time window between the estimated time-specific contact matrices. For example, when matrices $\boldsymbol{C^{asym}(t_1)}$ and $\boldsymbol{C^{asym}(t_2)}$ represent the social contact behaviour at time step t_1 and t_2, respectively, then $\boldsymbol{C^{asym}(t)}$ is calculated as follows:

$$\boldsymbol{C^{asym}(t)} = \begin{cases} \boldsymbol{C^{asym}(t_1)} & \text{if } t \in \ (t_1 + 5;\ t_2) \\ \left[1 - \frac{t-t_2}{5}\right] \boldsymbol{C^{asym}(t_1)} + \left[\frac{t-t_2}{5}\right] \boldsymbol{C^{asym}(t_2)} & \text{if } t \in \ [t_2;\ t_2 + 5] \\ \boldsymbol{C^{asym}(t_2)} & \text{if } t \in \ (t_2 + 5;\ t_3) \end{cases}$$

Table S1: Included social contact data in the transmission model: wave Id, start date, data source and additional wave Id* for the sensitivity analysis with multi-wave q-parameters.

Id	Start date	Data source	Id*
0	2020-03-01	BE 2010 Survey [4, 5]	0
1	2020-03-15	CoMix BE, wave 1 [6]	1
2	2020-04-01		2
3	2020-04-17		3
4	2020-05-08	CoMix BE, wave 2 [6]	3
5	2020-05-22	CoMix BE, wave 3 [6]	3
6	2020-06-04	CoMix BE, wave 4 [6]	3
7	2020-06-18	CoMix BE, wave 5 [6]	3
8	2020-07-02	CoMix BE, wave 6 [6]	3
9	2020-07-16	CoMix BE, wave 7 [6]	3
10	2020-07-30	CoMix BE, wave 8 [6]	3
11	2020-09-01	CoMix BE, wave 8 [6]	4
12	2020-09-15		5
13	2020-10-01		6
14	2020-10-20		7
15	2020-10-27		8
16	2020-11-03		9
17	2020-11-12	CoMix BE, wave 9 [2]	10
18	2020-11-27	CoMix BE, wave 10 [2]	10
19	2020-12-10	CoMix BE, wave 11 [2]	10
20	2020-12-22	CoMix BE, wave 12 [2]	11
21	2021-01-05	CoMix BE, wave 13 [2]	11
22	2021-01-19	CoMix BE, wave 14 [2]	11
23	2021-02-02	CoMix BE, wave 15 [2]	11
24	2021-02-16	CoMix BE, wave 16 [2]	11
25	2021-03-02	CoMix BE, wave 17 [2]	11
26	2021-03-16	CoMix BE, wave 18 [2]	12
27	2021-03-30	CoMix BE, wave 19 [2]	12
28	2021-04-13	CoMix BE, wave 20 [2]	12
29	2021-04-27	CoMix BE, wave 21 [2]	12
30	2021-05-12	CoMix BE, wave 22 [2]	12
31	2021-05-25	CoMix BE, wave 23 [2]	12
32	2021-06-09	CoMix BE, wave 24 [2]	12
33	2021-06-22	CoMix BE, wave 25 [2]	12
34	2021-07-06	CoMix BE, wave 26	12
35	2021-07-20	CoMix BE, wave 27	12
36	2021-08-03	CoMix BE, wave 28	12
37	2021-08-17	CoMix BE, wave 29	12
38	2021-08-31	CoMix BE, wave 30	12
39	2021-09-14	CoMix BE, wave 31	12
40	2021-09-28	CoMix BE, wave 32	13
41	2021-10-12	CoMix BE, wave 33	13
42	2021-10-27	CoMix BE, wave 34	13
43	2021-11-09	CoMix BE, wave 35	13
44	2021-11-23	CoMix BE, wave 36	13
45	2021-12-07	CoMix BE, wave 37	13
46	2021-12-21	CoMix BE, wave 38	13
47	2022-01-04	CoMix BE, wave 39	13
48	2022-01-18	CoMix BE, wave 40	13
50	2022-02-01	CoMix BE, wave 41	13
51	2022-02-16	CoMix BE, wave 42	13

S1.3 Immunity

The model structure in Figure S1 and previous differential equations account for vaccine- and infection-induced immunity. Individuals in an $\boldsymbol{R_i}$ compartment cannot be re-infected and parameters ν_i and ζ_i denote the vaccine-induced reduced susceptibility and severity of disease, respectively. To account for age-specific vaccine uptake ($\boldsymbol{\Omega}$) and waning immunity of infection- (π) and vaccine-induced (α) immunity, we duplicated the two-strain version, which is presented in Figure 2. The following set of ordinary differential equations describes the (deterministic version of the) transitions in the age- and time-specific Susceptible ($\boldsymbol{S(t)}$) and Recovered ($\boldsymbol{R_i(t)}$) classes with i representing either model strain a or b:

$$\begin{aligned}
\frac{d\boldsymbol{S^{naive}(t)}}{dt} &= -\left\{\boldsymbol{\Omega^{mRNA_1}(t)} + \boldsymbol{\Omega^{adeno_1}(t)}\right\}\boldsymbol{S^{naive}(t)}\\
\frac{d\boldsymbol{S^{mRNA_1}(t)}}{dt} &= \boldsymbol{\Omega^{mRNA_1}(t)}\left\{\boldsymbol{S^{naive}(t)} + \boldsymbol{S^{waning_1}(t)}\right\} - \boldsymbol{\Omega^{mRNA_2}(t)S^{mRNA_1}(t)}\\
\frac{d\boldsymbol{S^{mRNA_2}(t)}}{dt} &= \boldsymbol{\Omega^{mRNA_2}(t)S^{mRNA_1}(t)} - \left\{\boldsymbol{\Omega^{booster}(t)} + \alpha^{mRNA_2}\right\}\boldsymbol{S^{mRNA_2}(t)}\\
\frac{d\boldsymbol{S^{adeno_1}(t)}}{dt} &= \boldsymbol{\Omega^{adeno_1}(t)}\left\{\boldsymbol{S^{naive}(t)} + \boldsymbol{S^{waning_1}(t)}\right\} - \boldsymbol{\Omega^{adeno_2}(t)S^{adeno_1}(t)}\\
\frac{d\boldsymbol{S^{adeno_2}(t)}}{dt} &= \boldsymbol{\Omega^{adeno_2}(t)S^{adeno_1}(t)} - \left\{\boldsymbol{\Omega^{booster}(t)} + \alpha^{adeno_2}\right\}\boldsymbol{S^{adeno_2}(t)}\\
\frac{d\boldsymbol{S^{booster}(t)}}{dt} &= \boldsymbol{\Omega^{booster}(t)}\left\{\boldsymbol{S^{mRNA_2}(t)} + \boldsymbol{S^{adeno_2}(t)} + \boldsymbol{S^{waning_2}(t)} + \boldsymbol{S^{wanings_3}(t)}\right\} - \alpha^{booster}\boldsymbol{S^{booster}(t)}\\
\frac{d\boldsymbol{S^{waning_1}(t)}}{dt} &= \pi\boldsymbol{R_i^{naive}(t)} - \left\{\boldsymbol{\Omega^{mRNA_1}(t)} + \boldsymbol{\Omega^{adeno_1}(t)}\right\}\boldsymbol{S^{waning_1}(t)}\\
\frac{d\boldsymbol{S^{waning_2}(t)}}{dt} &= \pi\left\{\boldsymbol{R_i^{mRNA_2}(t)} + \boldsymbol{R_i^{adeno_2}(t)} + \boldsymbol{R_i^{booster}(t)} + \boldsymbol{R_i^{wanings_3}(t)} + \boldsymbol{R^{waning_4}(t)}\right\} - \alpha^{booster}\boldsymbol{S^{waning_2}(t)}\\
\frac{d\boldsymbol{S^{waning3}(t)}}{dt} &= \alpha^{mRNA_2}\left\{\boldsymbol{S^{mRNA_2}(t)} + \boldsymbol{S^{adeno_2}(t)}\right\} - \boldsymbol{\Omega^{booster}(t)S^{wanings_3}(t)}\\
\frac{d\boldsymbol{S^{waning4}(t)}}{dt} &= \alpha^{booster}\boldsymbol{S^{booster}(t)} - \boldsymbol{\Omega^{booster}(t)S^{waning4}(t)}
\end{aligned}$$

$$\begin{aligned}
\frac{d\boldsymbol{R_i^{naive}(t)}}{dt} &= -\left\{\boldsymbol{\Omega^{mRNA_1}(t)} + \boldsymbol{\Omega^{adeno_1}(t)}\right\}\boldsymbol{R_i^{naive}(t)}\\
\frac{d\boldsymbol{R_i^{mRNA_1}(t)}}{dt} &= \boldsymbol{\Omega^{mRNA_1}(t)}\left\{\boldsymbol{R_i^{naive}(t)} + \boldsymbol{R_i^{waning_1}(t)}\right\} - \boldsymbol{\Omega^{mRNA_2}(t)R_i^{mRNA_1}(t)}\\
\frac{d\boldsymbol{R_i^{mRNA_2}(t)}}{dt} &= \boldsymbol{\Omega^{mRNA_2}(t)R_i^{mRNA_1}(t)} - \left\{\boldsymbol{\Omega^{booster}(t)} + \alpha^{mRNA_2} + \pi\right\}\boldsymbol{R_i^{mRNA_2}(t)}\\
\frac{d\boldsymbol{R_i^{adeno_1}(t)}}{dt} &= \boldsymbol{\Omega^{adeno_1}(t)}\left\{\boldsymbol{R_i^{naive}(t)} + \boldsymbol{R_i^{waning_1}(t)}\right\} - \left\{\boldsymbol{\Omega^{adeno_2}(t)} + \pi\right\}\boldsymbol{R_i^{adeno_1}(t)}\\
\frac{d\boldsymbol{R_i^{adeno_2}(t)}}{dt} &= \boldsymbol{\Omega^{adeno_2}(t)R_i^{adeno_1}(t)} - \left\{\boldsymbol{\Omega^{booster}(t)} + \alpha^{adeno_2} + \pi\right\}\boldsymbol{R_i^{adeno_2}(t)}\\
\frac{d\boldsymbol{R_i^{booster}(t)}}{dt} &= \boldsymbol{\Omega^{booster}(t)}\left\{\boldsymbol{R_i^{mRNA_2}(t)} + \boldsymbol{R_i^{adeno_2}(t)} + \boldsymbol{R_i^{waning_2}(t)} + \boldsymbol{R_i^{wanings_3}(t)}\right\} - \left\{\alpha^{booster} + \pi\right\}\boldsymbol{R_i^{booster}(t)}
\end{aligned}$$

$$\frac{d\boldsymbol{R_i^{waning_1}(t)}}{dt} = -\left\{\boldsymbol{\Omega^{mRNA_1}(t)} + \boldsymbol{\Omega^{adeno_1}(t)} + \pi\right\}\boldsymbol{R_i^{waning_1}(t)}$$
$$\frac{d\boldsymbol{R_i^{waning_2}(t)}}{dt} = -\left\{\boldsymbol{\Omega^{booster}(t)} + \pi\right\}\boldsymbol{R_i^{waning_2}(t)}$$

$$\frac{d\boldsymbol{R_i^{waning3}(t)}}{dt} = \alpha^{mRNA_2}\left\{\boldsymbol{R_i^{mRNA_2}(t)} + \boldsymbol{R_i^{adeno_2}(t)}\right\} - \left\{\boldsymbol{\Omega^{booster}(t)} + \pi\right\}\boldsymbol{R_i^{wanings_3}(t)}$$
$$\frac{d\boldsymbol{R_i^{waning4}(t)}}{dt} = \alpha^{booster}\boldsymbol{R_i^{booster}(t)} - \left\{\boldsymbol{\Omega^{booster}(t)} + \pi\right\}\boldsymbol{R_i^{waning4}(t)}$$

with, e.g., $\boldsymbol{\Omega^{mRNA1}(t)}$ representing the age- and time-specific uptake of the first dose of the mRNA vaccine, α^{adeno_2} the waning immunity rate after two doses of an adeno-based vaccine and π the waning rate of infection-induced immunity.

S1.4 Discrete time stochastic epidemic model

The spread of the virus is hampered by reductions in the number of contacts and changes in the way contacts are made, either voluntarily or as a consequence of government intervention. These time- (and age-) dependent behavioural changes introduce substantial uncertainty in the subsequent course of the outbreak and require stochastic model components to evaluate the effectiveness of the intervention strategies and to make future predictions in terms of, for example, new hospitalisations. Moreover, stochastic epidemic models allow to determine the probability of extinction based on multiple realisations of the model. Therefore, we amended the deterministic model hitherto described into a discrete-time stochastic epidemic model to describe the transmission process under the mitigation strategies as highlighted hereabove.

Our chain binomial model, originally introduced by Bailey [7], is a so-called discrete-time stochastic alternative to the continuous-time deterministic model based on the health states and transitions presented in Figure S1. The chain-binomial model assumes a stochastic version of an epidemic obtained through a succession of discrete generations of infected individuals in a probabilistic manner. Consider a time interval $(t, t+h]$, where h represents the length between two consecutive time points at which we evaluate the model, here $h = 1/4$ day. Let us assume that there are $S(t,k)$ susceptible individuals at time t in age group k, we expect $S(t,k)p_i^*(t,k)$ newly exposed individuals in age group k by viral strain i at time $t+h$, i.e.,

$$E_i^{new}(t+h,k) \sim \text{Binomial}\left(S(t,k), p_i^*(t,k) = 1 - \{1 - p_i(t,k)\}^{\sum_{k'=1}^{K} I_i(t,k')}\right),$$

where $I_i(t,k')$ represent the number of infected individuals in age group k' by viral strain i at time t and $p_i(t,k)$ represents the transmission probability conditional upon contact between a susceptible individual in age group k and an infected individual by strain i. The probability that a susceptible individual escapes infection during a single contact with an infected individual by strain i is equal to $(1 - p_i(t,k))$. When assuming that all contacts are equally infectious, the escape probability is $(1 - p_i(t,k))^m$ in the case that the susceptible individual contacts m infectious individuals with strain i. In this setting, the probability of infection $p_i^*(t,k)$ for a susceptible individual in age group $k = 1, \dots K$ can be obtained as:

$$p_i^*(t,k) = 1 - \exp\left[-h\nu_i\tau_i \sum_{k'=1}^{K} \beta^{sym}(t,k,k')\left\{I^{mild}(t,k') + I^{severe}(t,k')\right\} + \beta^{asym}(t,k,k')\left\{I^{presym}(t,k') + I^{asym}(t,k')\right\}\right],$$

with h the step size, ν_i the vaccine-induced immunity against infection by strain i, τ_i the VOC-specific scaling factor related to the transmission potential relative to the original strain, $\beta^{sym}(t,k,k')$ the time-specific transmission parameter from a symptomatic infected individual of age k' to a susceptible individual of age k, and $\beta^{asym}(t,k,k')$ is the counterpart for the transmission from an asymptomatic individual of age k'.

The number of individuals in age group k leaving the exposed state (and entering the pre-symptomatic compartment) within the specified time interval is

$$I_i^{presym,new}(t+h,k) \sim \text{Binomial}\left(E_i(t,k), 1-\exp\left(-h\gamma_i\right)\right),$$

where $1/\gamma_i$ equals the mean length of the latency period of strain i. Probabilistic transitions in the other compartments are derived similarly, hence, a discrete age-structured stochastic model (with step size $h = 1/4$ day) with i representing strain a and b is fully specified by

$$\begin{aligned}
I_i^{asym,new}(t+h,k) &\sim \text{Binomial}\left(I_i^{presym}(t,k), 1-\exp\left(-hp(k)\theta\right)\right),\\
I_i^{mild,new}(t+h,k) &\sim \text{Binomial}\left(I_i^{presym}(t,k), 1-\exp\left(-h[1-p(k)]\theta\right)\right),\\
I_i^{sev,new}(t+h,k) &\sim \text{Binomial}\left(I_i^{mild}(t,k), 1-\exp\left(-h\zeta_i[1-\psi_i(k)]\delta_2\right)\right),\\
I_i^{hosp,new}(t+h,k) &\sim \text{Binomial}\left(I_i^{sev}(t,k), 1-\exp\left(-h\phi_i(k)\omega(t)\right)\right),\\
I_i^{icu,new}(t+h,k) &\sim \text{Binomial}\left(I_i^{sev}(t,k), 1-\exp\left(-h[1-\phi_i(k)]\omega(t)\right)\right),\\
D_i^{hosp,new}(t+h,k) &\sim \text{Binomial}\left(I_i^{hosp}(t,k), 1-\exp\left\{-h\mu_i(k)\delta_{3i}\right\}\right),\\
D_i^{icu,new}(t+h,k) &\sim \text{Binomial}\left(I_i^{icu}(t,k), 1-\exp\left\{-h\mu_i(k)\delta_{3i}\right\}\right),\\
R_i^{asym,new}(t+h,k) &\sim \text{Binomial}\left(I_i^{asym}(t,k), 1-\exp\left(-h\delta_1\right)\right),\\
R_i^{mild,new}(t+h,k) &\sim \text{Binomial}\left(I_i^{mild}(t,k), 1-\exp\left(-h\phi_i(k)\delta_2\right)\right),\\
R_i^{hosp,new}(t+h,k) &\sim \text{Binomial}\left(I_i^{hosp}(t,k), 1-\exp\left[-h\delta_{3i}\left\{1-\mu_i(k)\right\}\right]\right),\\
R_i^{icu,new}(t+h,k) &\sim \text{Binomial}\left(I_i^{icu}(t,k), 1-\exp\left[-h\delta_{3i}\left\{1-\mu_i(k)\right\}\right]\right),
\end{aligned}$$

and

$$\begin{aligned}
S(t+h,k) &= S(t,k) - E_a^{new}(t+h,k) - E_b^{new}(t+h,k),\\
E_i(t+h,k) &= E_i(t,k) + E_i^{new}(t+h,k) - I_i^{presym,new}(t+h,k),\\
I_i^{presym}(t+h,k) &= I_i^{presym}(t,k) + I_i^{presym,new}(t+h,k) - I_i^{asym,new}(t+h,k) - I_i^{mild,new}(t+h,k),\\
I_i^{asym}(t+h,k) &= I_i^{asym}(t,k) + I_i^{asym,new}(t+h,k) - R_i^{asym,new}(t+h,k),\\
I_i^{mild}(t+h,k) &= I_i^{mild}(t,k) + I_i^{mild,new}(t+h,k) - I_i^{sev,new}(t+h,k) - R_i^{mild,new}(t+h,k),\\
I_i^{sev}(t+h,k) &= I_i^{sev}(t,k) + I_i^{sev,new}(t+h,k) - I_i^{hosp,new}(t+h,k) - I_i^{icu,new}(t+h,k),\\
I_i^{hosp}(t+h,k) &= I_i^{hosp}(t,k) + I_i^{hosp,new}(t+h,k) - D_i^{hosp,new}(t+h,k) - R_i^{hosp,new}(t+h,k),\\
I_i^{icu}(t+h,k) &= I_i^{icu}(t,k) + I_i^{icu,new}(t+h,k) - D_i^{icu,new}(t+h,k) - R_i^{icu,new}(t+h,k),\\
D_i(t+h,k) &= D_i(t,k) + D_i^{hosp,new}(t+h,k) + D_i^{icu,new}(t+h,k),\\
R_i(t+h,k) &= R_i(t,k) + R_i^{asym,new}(t+h,k) + R_i^{mild,new}(t+h,k) + R_i^{hosp,new}(t+h,k) + R_i^{icu,new}(t+h,k).
\end{aligned}$$

Results based on the stochastic discrete time age-structured epidemic model account for two sources of variability, namely (1) variability coming from the observational process reflected in uncertainty about the model parameters; and (2) variability introduced by the stochastic process. An overview of the parameter values and distributional assumptions are listed in Tables S3.

S2 Vaccine uptake

The age-specific vaccine uptake, by dose and vaccine-type, in the dynamic model is based on the reported uptake for Belgium. Figure S2 presents the reported uptake, except the the second booster, by age group, which was used as baseline in the scenario analysis. Figures S3 present the altered vaccine uptake for the different scenarios.

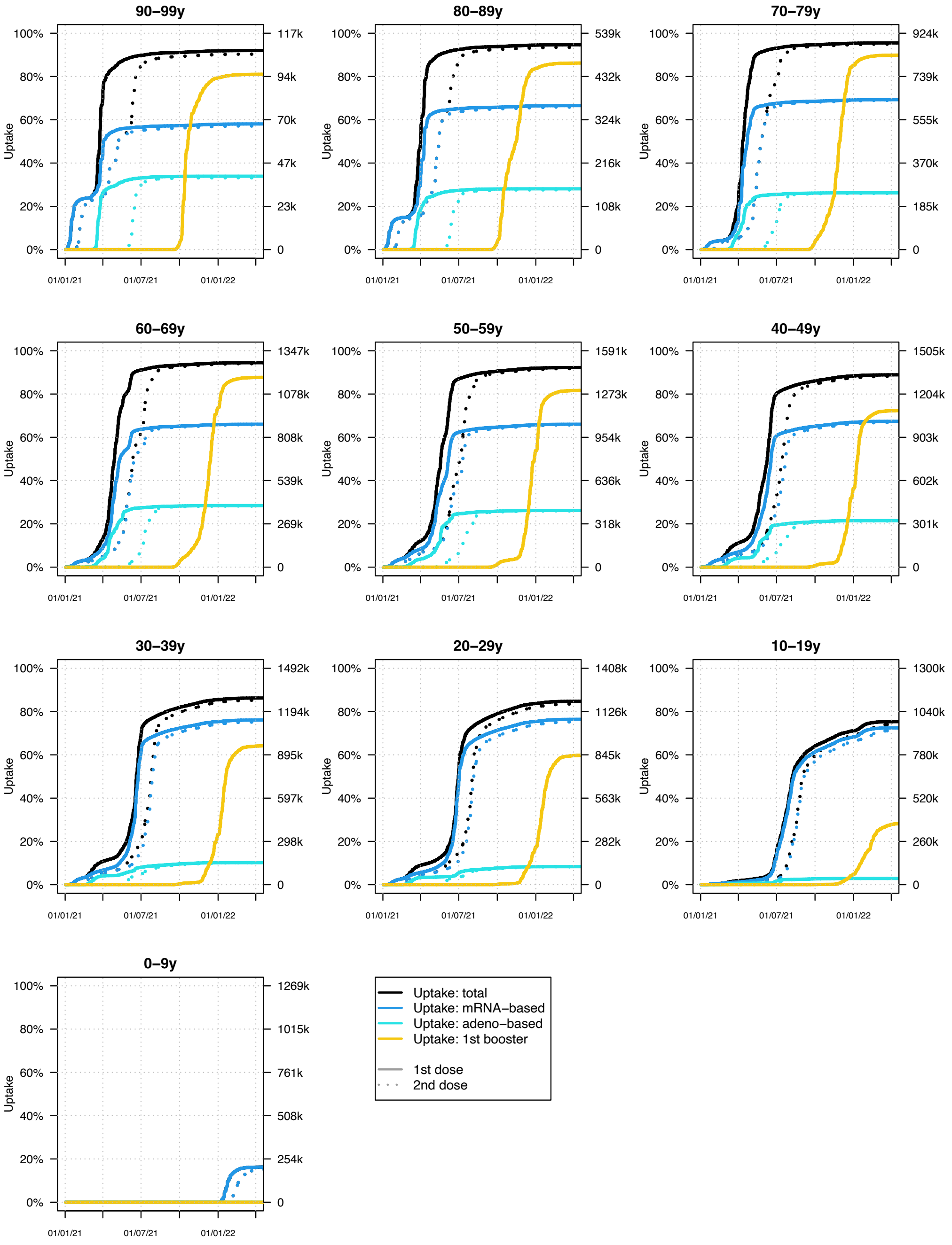

Figure S2: Vaccine uptake by age, vaccine-type and dose based on the reported uptake for Belgium.

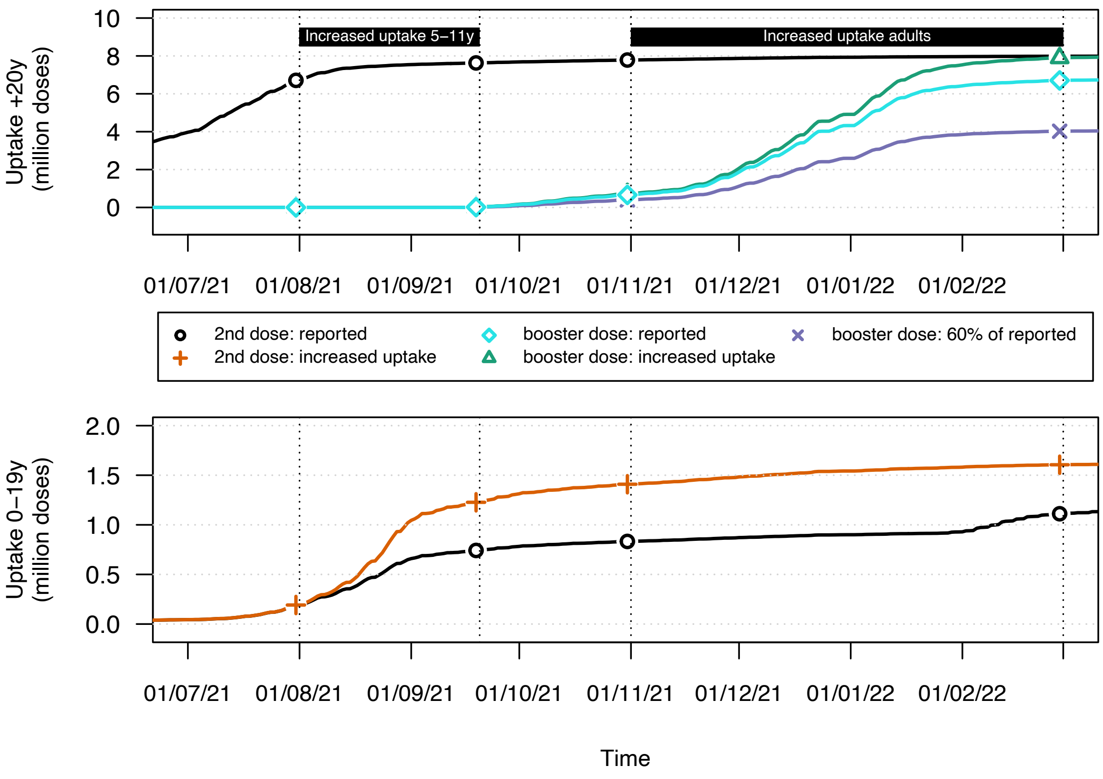

Figure S3: Reported and scenario-based uptake of COVID-19 vaccines over time in terms of second and first booster doses in adults above the age of 20 years (top) and among 0-19 year old minors (bottom).

S3 Model calibration

In this section we briefly describe the parameter estimation procedure and different data sources that are considered to fit the models.

Next to the social contact data used to inform the transmission parameters, five different data sources were used to fit the stochastic compartmental model, namely (1) incidence data on the daily number of new hospitalisations; (2) serial serological survey data collected during the initial phase of the epidemic [?]; (3) baseline genomic surveillance data of SARS-CoV-2 in Belgium; (4) prevalence data on hospitalised COVID-19 cases in general and in ICU (i.e., the total number of hospitalised and ICU admitted patients at a specific moment in time); and (5) incidence data on the daily number of new deaths. The following distributional assumptions are made with regard to the different outcome variables:

$$Y(t+1,k) = \sum_{j=1}^{4} Y(t+jh,k) \sim \text{Binomial}\left(\sum_{i=a}^{b}\sum_{j=1}^{4} I_i^{sev}(t+\{j-1\}h,k)\ , 1-\exp\left(-h\omega(k)\right)\right),$$

$$W(t^*,k) \sim \text{Binomial}\left(n(t^*,k), \pi(t^*,k) = \frac{1}{N(k)}\sum_{t=0}^{t^*} p_{sens}(t^*-t)\sum_{i=a}^{b}\{I_i^{presym}(t,k) + I_i^{asym}(t,k)\}\right),$$

$$U(t+1) = \sum_{j=1}^{4} U(t+jh) \sim \text{Binomial}\left(m(t), \sum_{j=1}^{4}\sum_{k=1}^{K}\frac{I_b^{mild}(t,k)}{\sum_{i=a}^{b} I_i^{mild}(t,k)}\right),$$

$$Z(t+1,k) = \sum_{j=1}^{4} Z(t+jh,k) \sim \text{Binomial}\left(\sum_{i=a}^{b}\sum_{j=1}^{4} I_i^{hosp}(t+\{j-1\}h,k) + I_i^{icu}(t+\{j-1\}h,k)\ , \sum_{i=a}^{b}\sum_{j=1}^{4}\frac{D_i(t+\{j-1\}h,k)}{I_i^{hosp}(t+\{j-1\}h,k) + I_i^{icu}(t+\{j-1\}h,k)}\right),$$

where $Y(t,k)$ represent the number of new hospitalisations at time t in age group k, respectively. Since we do not have data on referral within hospitals, we do not explicitly distinguish between hospitalised and ICU admitted patients in terms of hospital discharge (including death), although the model is equipped to do so. $W(t^*,k)$ represents the total number of seropositive individuals in age group k in a cross-sectional serological collection of residual blood samples performed at time t^*. All individuals tested in age group k at time t^*, denoted by $n(t^*,k)$, have a probability $\pi(t^*,k)$ (i.e., equal to the observed seroprevalence) to be classified as seropositive accounting for sensitivity of the test $p_{sens}(t_o)$ as a function of time since symptom onset and assuming perfect specificity of the test. The sensitivity of the test is assumed to follow a logistic growth curve based on available information in the literature [8]. For more details, the reader is referred to Abrams et al. (2021) [1]. $U(t)$ represents the total number of individuals of the baseline genomic surveillance of SARS-CoV-2 in Belgium by the National Reference Laboratory that match the "b" strain of the model at time t. The total number of individuals included in the surveillance at time t is denoted by $m(t)$. $Z(t,k)$ represents the number of new deaths at time t in age group k. In the absence of data on deaths of ICU and general hospital ward admitted patients, we rely on age-specific mortality rates for hospitalised patients in general. In addition, we could not distinguishing VOC-specific mortality in the reference data to estimate VOC-specific mortality parameters. As such, we included the model-based case-fatality ratio in our estimation procedure.

Lacking timely and VOC-specific data on hospital discharges of ICU and general hospital ward admitted patients, in combination with within- and between-hospital referrals and dependence of hospital load data over time, we did not consider a (conditional) binomial distribution to model the hospital occupancy data. More specifically, model parameters related to hospital load (hence, (average) length of stay in the hospital)

were not estimated using a likelihood-based approach. As such, we opted to use a weighted sum of squares approach to age-specific model parameters related to hospital load in total and specifically in ICU:

$$WLS_{hospital} = \sum_{t=1}^{T} \sum_{k=1}^{K} \left(\frac{Q(t,k) - \sum_{i=a}^{b} \left\{ I_i^{hosp}(t,k) + I_i^{icu}(t,k) \right\}}{\sum_{i=a}^{b} \left\{ I_i^{hosp}(t-1,k) + I_i^{icu}(t-1,k) \right\}} \right)^2,$$

$$WLS_{icu} = \sum_{t=1}^{T} \sum_{k=1}^{K} \left(\frac{V(t,k) - \sum_{i=a}^{b} I_i^{icu}(t,k)}{\sum_{i=a}^{b} I_i^{icu}(t,k)} \right)^2,$$

with $Q(t,k)$ and $V(t,k)$ represent the overall hospital load and ICU load, respectively, for age group k at time point t.

We defined two criteria using importance weights when combining the daily hospital admission data with the two age-specific serology data points:

$$\text{Crit}_1 = \sum_{k=1}^{K} \left(\sum_{t=1}^{T} Y(t,k) + \sum_{t^*=1}^{2} \frac{T}{K} \frac{w(t^*,k)}{\sum_{k=1}^{K} w(t^*,k)} W(t^*,k) \right),$$

$$\text{Crit}_2 = LL_1 + \sum_{t=1}^{T} U(t),$$

with N_k the population size per age cohort and

$$w(t^*,k) = \frac{N_k}{n(t^*,k)}$$

In order to capture the hospital load-related parameters, the following criterion was considered:

$$\text{Crit}_3 = -WLS_{hospital} - WLS_{icu}.$$

Fourthly, the multi-step parameter estimation procedure continued with the estimation of the mortality-related parameters:

$$\text{Crit}_4 = \sum_{k=1}^{K} \sum_{t=1}^{T} Z(t,k).$$

S3.1 Model initialisation

The number of imported cases (and first generation(s) of infected cases through local transmission) is determined from the age-specific number of confirmed cases on 12 March 2020. More specifically, given a number $n_0(k)$ of confirmed cases in age group k, the expected number of imported cases in age class k equals

$$n_0(k) \left(\frac{1}{1 - p(k)} \right),$$

where $p(k)$ represents the asymptomatic fraction in age group k thereby assuming that confirmed cases solely reflect the proportion of mildly and severely ill individuals. The introduction of the imported cases in the system is presumed to take place on 1 March following the school holiday period.

S3.2 Estimation

We used Bayesian methods to fit our transmission model to multiple data sources including daily hospital admissions and bed occupancy, early seroprevalence, genomic surveillance and mortality. The calibration procedure relies on series of MCMC sampling resulting in 10 posterior samples of the joint distribution. Model parameters related to hospital incidence, VOC prevalence and mortality have been estimated using a likelihood approach based on a binomial distribution (see above). The MCMC procedure was based on the adaptive Metropolis-within-Gibbs algorithm, and parameter configurations were updated starting from previous calibration results. Each MCMC procedure consisted of an additional number of iterations (see Table S2) with 10 realisations per iteration, periodicity of 10 iterations and leading to 60 different chains based on 60 initial starting configurations. For the scenario analysis, we selected the 10 best scoring chains.

In the absence of age-specific and daily data on hospital discharges and transition between the general ward and ICU, we informed the model by the reported hospital bed occupancy for COVID-19 in general and in ICU. This prevalence-based approach could not adopt a likelihood so we applied the MCMC procedure based on least squares scoring weighted by the daily reported load to estimate parameters characterising the hospital load in general and in ICU.

Many model parameters have a time-specific impact on the model or are correlated. Therefore we opted for a sequential parameters estimation process with the parameters and time horizon presented in Table S2.

Table S2: Summary of the step-wise parameter estimation procedure with subscript i referring to the SARS-CoV-2 variant and e.g. $\boldsymbol{q^0}$ to the age-specific proportionality factors for period Id=0 as listed in Table S1.

**Step ID**	**Time horizon**	**Included parameters (#)**	**Objective function**	**MCMC Iterations***	**Run Time**
Wave 1	120 days (Jun 28th 2020)	γ, θ, δ_1, δ_2, $\boldsymbol{\phi_0}$, $\boldsymbol{\omega}$, $\boldsymbol{q^0}$, n^0, $\boldsymbol{q^{1-5}}$ (76)	Crit_1	3000	24h
Wave 2	305 days (Dec 30th 2020)	$\boldsymbol{q^{5-19}}$ (150)	Crit_1	1000	36h
Alpha VOC	457 days (May 31st 2021)	n^0_{alpha}, τ_{alpha}, $\boldsymbol{q^{19-28}}$ (102)	Crit_2	500	31h
Delta VOC	563 days (Sep 14th 2021)	n^0_{delta}, τ_{delta}, $\boldsymbol{q^{28-38}}$ (112)	Crit_2	400	38h
September 2021	650 days (Dec 10th 2021)	$\boldsymbol{q^{38-44}}$ (70)	Crit_2	400	31h
Omicron VOC (B.A.1 and B.A.2)	730 days (Feb 28th 2022)	$n^0_{omicron}$, $\tau_{omicron}$, $\gamma_{omicron}$, $\boldsymbol{q^{44-51}}$ (73)	Crit_2	400	38h
Hospital load	730 days (Feb 28th 2022)	$\delta_{3i}, \boldsymbol{\phi_i}$ (11)	Crit_3	1000	14h
Mortality	730 days (Feb 28th 2022)	$\boldsymbol{\mu_i}$ (40)	Crit_4	400	22h

*Each MCMC chain started from a random set of initial parameter values (max 10%) starting from previous parameter estimates and were manually checked with respect to MCMC mixing and convergence.

S3.3 Overview of model parameters

An overview of the (estimated) model parameters related to SARS-CoV-2/COVID-19 transmission dynamics are provided in Table S3 and S4.

Table S3: List of time invariant model parameters.

Notation	Description	Value	Prior distribution
θ^{-1}	Average length of pre-symptomatic infectious period	2.3 [1.4;3.3] days	$\theta \sim N(1/2, 0.05^2)$
$\boldsymbol{\omega}^{-1}$	Average time between symptom onset and hospitalisation, by age group	5.21 [0.58; 6.94] days,<br>6.7 [6.3; 7.0] days,<br>6.5 [5.3; 7.0] days,<br>6.9 [6.6; 7.0] days,<br>6.4 [5.5; 7.0] days,<br>4.7 [3.3; 5.8] days,<br>4.5 [2.9;5.7] days,<br>2.2 [1.3; 3.2] days,<br>1.7 [1.0; 2.2] days,<br>0.68 [0.51; 1.32] days,	$\boldsymbol{\omega} \sim U(1/7, 2)$
δ_1^{-1}	Average length of infectious period when asymptotically infected (after pre-symptomatic phase)	2.2 [1.7; 2.7] days	$\delta_1 \sim N(1/3.5, 0.05^2)$
δ_2^{-1}	Average length of infectious period when mildly infected (after pre-symptomatic phase)	4.5 [2.9; 6.3] days	$\delta_2 \sim N(1/7, 0.05^2)$
$\boldsymbol{p}$	Proportion fully asymptomatic cases, by age	0.6, 0.6, 0.49, 0.49, 0.32, 0.32, 0.34, 0.34, 0.34, 0.34	Fixed
h	Resolution of the binomial chain	0.25 day	Model choice
r	Relative infectiousness of asymptomatic vs. symptomatic cases	0.51	Fixed

Table S4: Estimated temporal, variant, and age-specific model parameters. The table should be interpreted from left to right, and empty boxes indicate that the parameters have not changed at that moment in time.

Notation	Description	Value 1	Value 2	Value 3	Value 4	Value 5	Value 6
t	Start date	2020-03-01	2020-09-01	2020-11-26	2021-05-10	2021-10-01	2021-11-25
i	Variant	wild-type		Alpha VOC	Delta VOC		Omicron VOC
	Initial cases	3131 [1392;4982]		3755 [2553;4899]	588 [35;1662]		266 [26;1065]
$1-\boldsymbol{\psi_i}$	Hospital hazard ratio, given symptomatic infection, by age	0.005 [0.004;0.007],<br>0.003 [0.002;0.004],<br>0.006 [0.005;0.007],<br>0.014 [0.013;0.016],<br>0.021 [0.018;0.024],<br>0.032 [0.027;0.038],<br>0.082 [0.065;0.101],<br>0.13 [0.11;0.15],<br>0.17 [0.13;0.19],<br>0.17 [0.14;0.21]			0.066 [0.051;0.081],<br>0.007 [0.006;0.009],<br>0.014 [0.012;0.015],<br>0.032 [0.029;0.036],<br>0.047 [0.040;0.053],<br>0.073 [0.061;0.085],<br>0.19 [0.15;0.23],<br>0.30 [0.25;0.35],<br>0.38 [0.30;0.43],<br>0.39 [0.32;0.47]		0.022 [0.017;0.027],<br>0.006 [0.005;0.008],<br>0.009 [0.008;0.010],<br>0.018 [0.017;0.021],<br>0.025 [0.022;0.029],<br>0.032 [0.027;0.038],<br>0.082 [0.065;0.101],<br>0.13 [0.11;0.15],<br>0.18 [0.15;0.21],<br>0.19 [0.16;0.23]
$1-\boldsymbol{\phi_i}$	Proportion severe infected cases that require ICU admission, by age	0.003 [0.003;0.003],<br>0.003 [0.003;0.003],<br>0.10 [0.099;0.102],<br>0.10 [0.099;0.102],<br>0.19 [0.19;0.19],<br>0.19 [0.19;0.19],<br>0.22 [0.22;0.22],<br>0.27 [0.26;0.27],<br>0.21 [0.21;0.21],<br>0.21 [0.21;0.21]		0.03 [0.03;0.04],<br>0.03 [0.03;0.04],<br>0.22 [0.22;0.23],<br>0.22 [0.22;0.23],<br>0.30 [0.30;0.31],<br>0.30 [0.30;0.31],<br>0.33 [0.32;0.33],<br>0.36 [0.36;0.36],<br>0.32 [0.32;0.32],<br>0.32 [0.32;0.32]	0.04 [0.02;0.05],<br>0.04 [0.02;0.05],<br>0.23 [0.17;0.24],<br>0.23 [0.17;0.24],<br>0.31 [0.26;0.32],<br>0.31 [0.26;0.32],<br>0.33 [0.29;0.34],<br>0.36 [0.33;0.37],<br>0.32 [0.28;0.33],<br>0.32 [0.28;0.33]	0.046 [0.0001;0.29],<br>0.046 [0.0001;0.29],<br>0.14 [0.01;0.42],<br>0.14 [0.01;0.42],<br>0.20 [0.04;0.44],<br>0.20 [0.04;0.44],<br>0.22 [0.06;0.45],<br>0.26 [0.09;0.46],<br>0.22 [0.05;0.50],<br>0.22 [0.05;0.50]	0.0002 [0.0002;0.0002],<br>0.0002 [0.0002;0.0002],<br>0.037 [0.035;0.042],<br>0.037 [0.035;0.042],<br>0.10 [0.10;0.11],<br>0.10 [0.10;0.11],<br>0.13 [0.13;0.14],<br>0.18 [0.18;0.19],<br>0.12 [0.12;0.13],<br>0.12 [0.12;0.13]
$\boldsymbol{\mu_i}$	Hospital fatality ratio, by age	0 [0;0],<br>3.5e-4 [0;2.4e-3],<br>3.5e-4 [0;2.4e-3],<br>0.024 [0.022;0.026],<br>0.033 [0.030;0.036],<br>0.080 [0.076;0.086],<br>0.20 [0.19;0.21],<br>0.43 [0.41;0.44],<br>0.85 [0.79;1.00],<br>1 [1;1]	0 [0;0],<br>3.0e-4 [0;4.3e-3],<br>3.0e-4 [0;4.3e-3],<br>0.016 [0.015;0.019],<br>0.033 [0.030;0.039],<br>0.062 [0.059;0.065],<br>0.14 [0.13;0.14],<br>0.23 [0.22;0.24],<br>0.48 [0.47;0.49],<br>0.98 [0.93;1.00]	0 [0;0],<br>1.1e-3 [0;7.1e-3],<br>1.1e-3 [0;7.1e-3],<br>0.007 [0.006;0.009],<br>0.015 [0.013;0.020],<br>0.036 [0.033;0.038],<br>0.105 [0.097;0.113],<br>0.20 [0.19;0.21],<br>0.25 [0.24;0.27],<br>0.25 [0.23;0.28]	0 [0;0],<br>6.3e-5 [0;9.0e-4],<br>6.3e-5 [0;9.0e-4],<br>0.009 [0.008;0.011],<br>0.028 [0.025;0.032],<br>0.053 [0.049;0.057],<br>0.11 [0.10;0.11],<br>0.17 [0.16;0.17],<br>0.22 [0.21;0.23],<br>0.37 [0.34;0.42]		0 [0;0],<br>1.4e-5 [0;6.4e-5],<br>1.4e-5 [0;6.4e-5],<br>0.0041 [0.0031;0.0074],<br>0.0090 [0.0063;0.0152],<br>0.040 [0.034;0.048],<br>0.069 [0.062;0.076],<br>0.090 [0.085;0.100],<br>0.11 [0.11;0.12],<br>0.19 [0.18;0.21]
δ_3^{-1}	Hospital length of stay	12 [12;12] days	11 [10;11]	10.1 [9.9;10.3] days	9.6 [9.2;11.2] days		6.7 [6.5;7.5] days
τ_i	Transmission advantage related to previous strain			0.32 [0.24;0.40]	0.98 [0.75;1.36]		0.41 [0.05;2.78]
γ_i^{-1}	Average length of latency period	1.7 [1.4;2.3] days					0.25 [0;1.2] days

S4 Estimated burden of disease

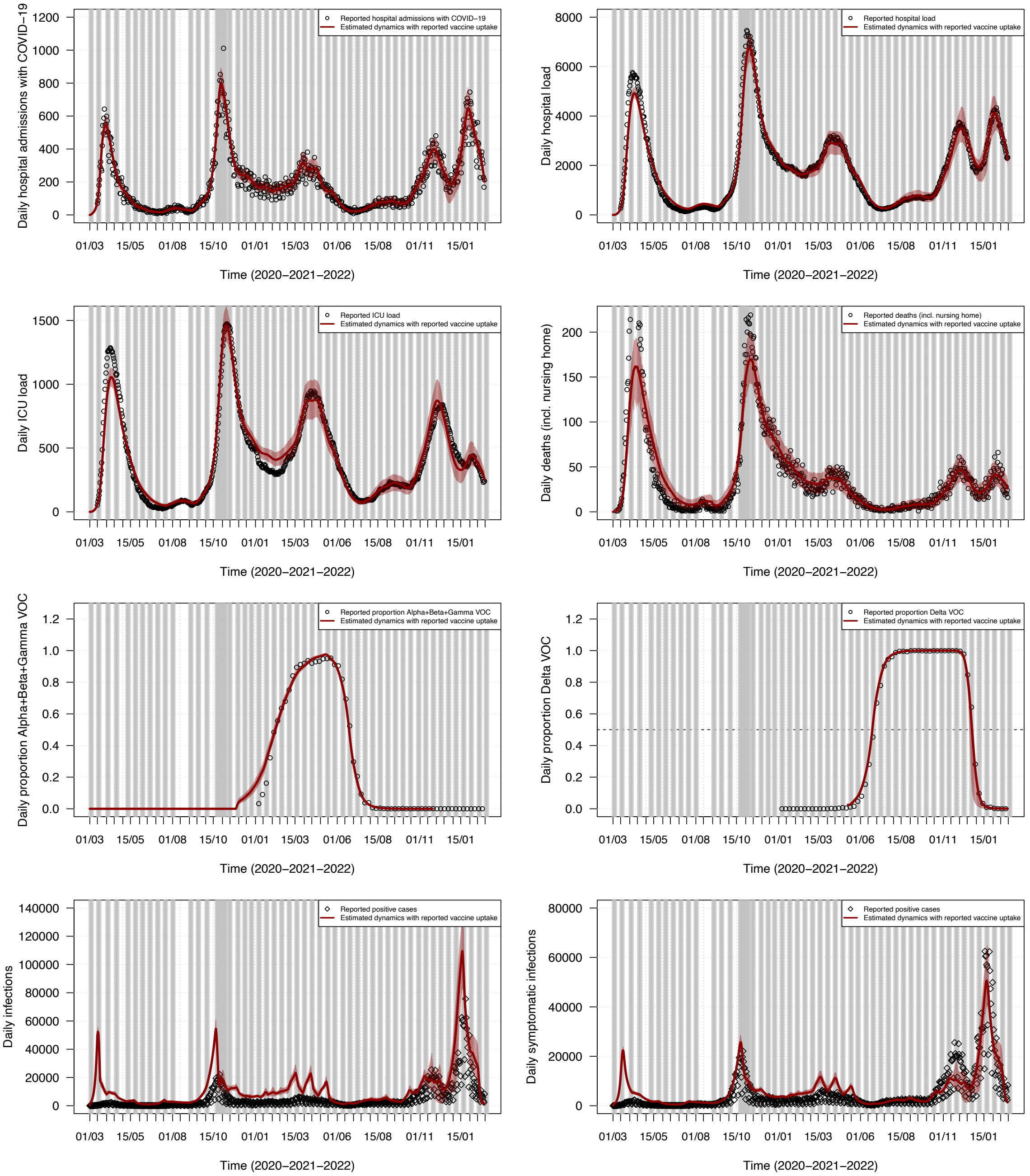

Figure S4: Model projections on the daily hospital admissions, hospital load, ICU load, disease related mortality, VOC-prevalence and daily number of (symptomatic) infections.

S5 Proportionality factors

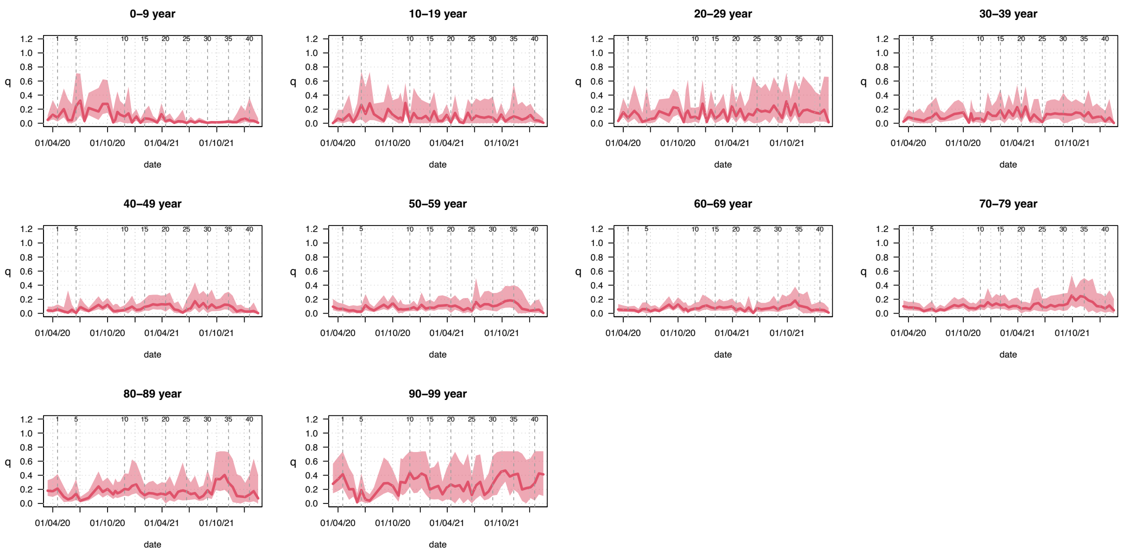

Figure S5: Estimated single-wave proportionality factors q by age and CoMix wave for the dynamic transmission model (default model configuration).

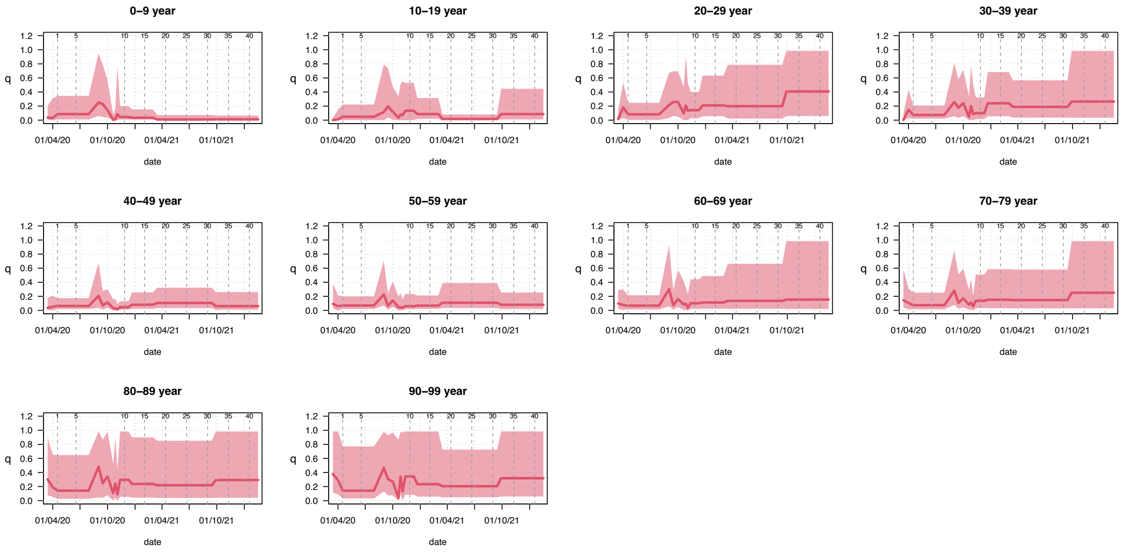

Figure S6: Estimated multi-wave proportionality factors q by age and CoMix wave for the dynamic transmission model (sensitivity analysis).

S6 Additional results

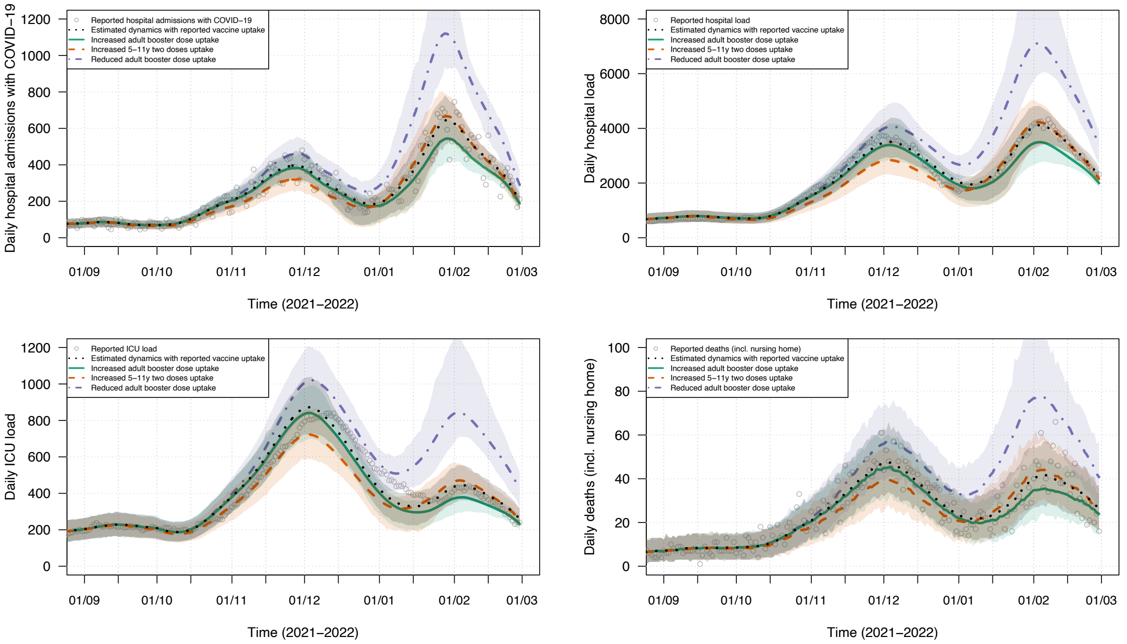

Figure S7: Effect of adjusted vaccine uptake on the COVID-19-related daily hospital admissions, hospital load, ICU load and mortality.

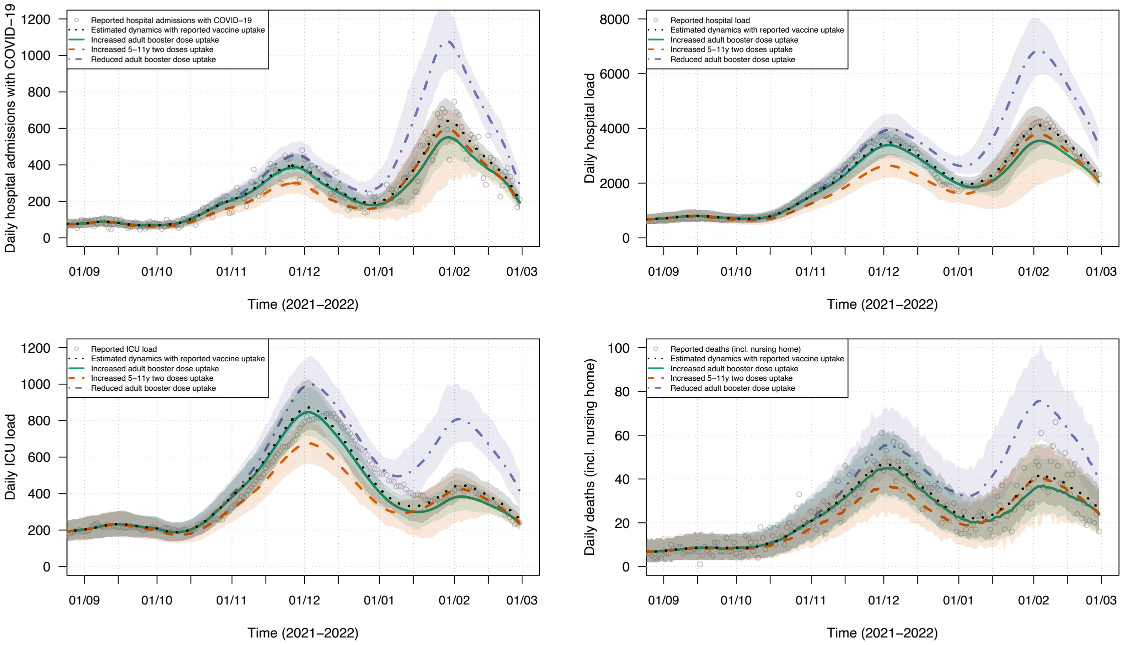

Figure S8: Effect of adjusted vaccine uptake assuming a vaccine-related protection against transmission of 30% on the COVID-19-related daily hospital admissions, hospital load, ICU load and mortality.

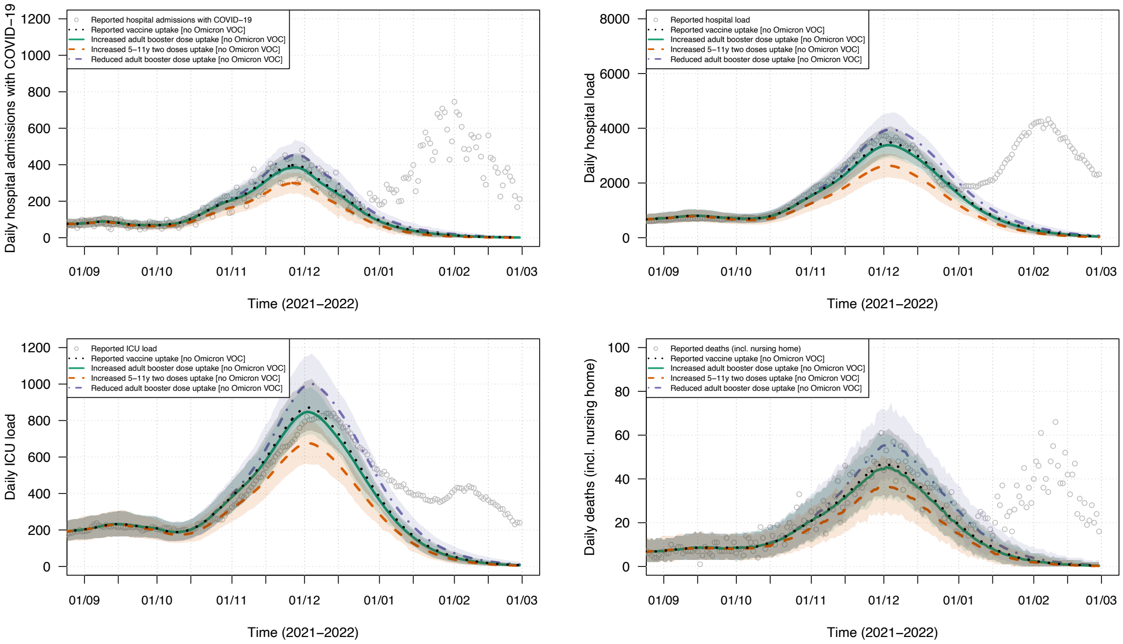

Figure S9: Model results assuming the absence of Omicron VOC and the effect of adjusted vaccine uptake on the projected hospital admissions and ICU load.

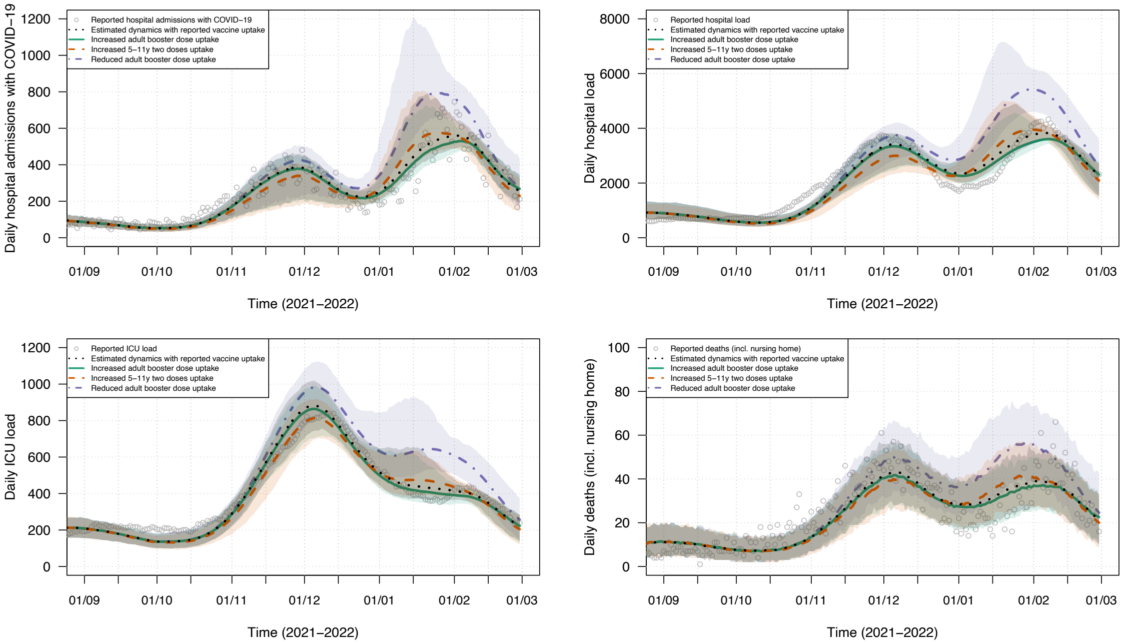

Figure S10: Model results based on aggregated proportionality factors (q) and the effect of adjusted vaccine uptake on the projected hospital admissions and ICU load.

Table S5: Projected burden of disease for children between 0-9 years of age between March 2020 and February 2022 and the incremental burden of disease for the vaccine uptake scenarios under study. The table presents the reported and incremental vaccine uptake and results are provided in terms of means and corresponding 95% credible intervals. Negative incremental effects within the credible intervals may arise as a result of altered stochastic paths between the baseline and intervention under study.

	**Burden of disease** from March 2020 until Feb 2022	**Incremental results with increased adult booster dose uptake**	**Incremental results with increased vaccine uptake 5-11y in 2021**	**Incremental results with reduced adult booster dose uptake**
**Total Infections**	1,195,672 [896,203;1,615,280]	-53,120 [-118,302;3,697]	-62,509 [-173,317;14,238]	+112,164 [41,795;248,379]
**Mild infections**	473,025 [353,060;642,394]	-20,932 [-47,064;1,510]	-23,723 [-66,566;6,234]	+44,173 [16,795;98,398]
**Hospital admissions**	5,874 [5,475;6,331]	-340 [-747;75]	-1,251 [-1,704;-783]	+773 [270;1,524]
**ICU admissions**	43 [27;59]	-1 [-12;10]	-9 [-26;8]	+1 [-11;13]
**Deaths**	0 [0;0]	0 [0;0]	0 [0;0]	0 [0;0]
**Vaccine Uptake (doses)**	25,671,309	+1,227,329	+905,113	-2,896,104

S7 References (Supplementary Information)

[1] Abrams, S., Wambua, J., Santermans, E., Willem, L., Kylen, E., Coletti, P., Libin, P., Faes, C., Petrof, O., Herzog, S.A., Beutels, P., Hens, N.: Modeling the early phase of the Belgian COVID-19 epidemic using a stochastic compartmental model and studying its implied future trajectories. Epidemics **35**, 100449 (2021)

[2] Verelst, F., Hermans, L., Vercruysse, S., Gimma, A., Coletti, P., Backer, J.A., Wong, K.L., Wambua, J., van Zandvoort, K., Willem, L., *et al.*: SOCRATES-CoMix: a platform for timely and open-source contact mixing data during and in between COVID-19 surges and interventions in over 20 European countries. BMC medicine **19**(1), 1–7 (2021)

[3] Van Kerckhove, K., Hens, N., Edmunds, W.J., Eames, K.T.D.: The impact of illness on social networks: implications for transmission and control of influenza. American Journal of Epidemiology **178**(11), 1655–1662 (2013). doi:10.1093/aje/kwt196

[4] Van Hoang, T., Coletti, P., Kiffe, Y.W., Van Kerckhove, K., Vercruysse, S., Willem, L., Beutels, P., Hens, N.: Close contact infection dynamics over time: insights from a second large-scale social contact survey in Flanders, Belgium, in 2010-2011. BMC Infectious Diseases **21**(1), 1–15 (2021)

[5] Willem, L., Van Kerckhove, K., Chao, D.L., Hens, N., Beutels, P.: A nice day for an infection? Weather conditions and social contact patterns relevant to influenza transmission. PLoS One **7**(11), 48695 (2012)

[6] Coletti, P., Wambua, J., Gimma, A., Willem, L., Vercruysse, S., Vanhoutte, B., Jarvis, C.I., van Zandvoort, K., Edmunds, J., Beutels, P., Hens, N.: CoMix: comparing mixing patterns in the Belgian population during and after lockdown. Sci Rep **10**(1), 21885 (2020)

[7] Bailey, N.T.J.: The Mathematical Theory of Infectious Diseases and Its Applications. Griffin, London (1975)

[8] Lou, B., Li, T., Zheng, S., Su, Y., Li, Z., Liu, W., Yu, F., Ge, S., Zou, Q., Yuan, Q., Lin, S., Hong, C., Yao, X., Zhang, X., Wu, D., Zhou, G., Hou, W., Li, T., Zhang, Y., Zhang, S., Fan, J., Zhang, J., Xia, N., Chen, Y.: Serology characteristics of SARS-CoV-2 infection since the exposure and post symptoms onset. European Respiratory Journal **57**(2) (2020)
